# DrugForm-DTA: Towards real-world drug-target binding affinity model

**DOI:** 10.1016/j.csbj.2025.09.023

**Published:** 2025-09-18

**Authors:** Ivan Khokhlov, Anna Tashchilova, Nikolai Bugaev-Makarovskiy, Olga Glushkova, Vladimir Yudin, Anton Keskinov, Sergey Yudin, Dmitry Svetlichnyy, Veronika Skvortsova

**Affiliations:** a Federal State Budgetary Institution “Centre for Strategic Planning and Management of Biomedical Health Risks” of the Federal Medical Biological Agency (Centre for Strategic Planning of FMBA of Russia), Pogodinskaya Street,10, bld. 1, Moscow 119121, Russia; b The Federal Medical Biological Agency (FMBA of Russia), Volokolamskoye Shosse, bld. 30, Moscow 123182, Russia

**Keywords:** Drug-target affinity prediction, DTA, Drug Discovery, QSAR, Deep learning, BindingDB

## Abstract

Drug-target affinity (DTA) prediction is a fundamental challenge in drug discovery. Computational methods for predicting DTA can greatly assist drug design by narrowing the search space and reducing the number of protein-ligand complexes with low affinity. Currently DTA approaches often do not require three-dimensional (3D) structural information of proteins, which is frequently unavailable. In this study we present the DrugForm-DTA model, which uses only structure-less representations of ligand and protein. It is a Transformer-based neural network with protein encoding based on ESM-2, and small molecule ligand encoding obtained with Chemformer. We evaluated the model on the standard benchmarks Davis and KIBA, and revealed superior performance of DrugForm-DTA with the best result for KIBA. Moreover, we developed a ready-to-use model trained on the BindingDB dataset which was subjected to high-quality filtering and transformation. Overall, our method predicts drug-target affinity values with a confidence level comparable to that of a single *in vitro* experiment. Also, we compared DrugForm-DTA against molecular modeling methods and revealed higher efficacy of the developed model for drug-target affinity predictions. Our investigation provides a high accuracy neural network model with performance comparable to that of experimental measurements, a filtered.and reassessed BindingDB dataset for further usage, and demonstrates the outstanding applicability of the proposed method for DTA prediction.

## Highlights

• Simple and effective neural network architecture to solve drug-target binding affinity problem
• The ready-to-train dataset based on the BindingDB database.
• A highly accurate DTA model, capable of solving real drug-design tasks.
• Our model outperforming existing methods and molecular modeling approaches in terms of binding affinity prediction.

## Graphical abstract

**Figure:**
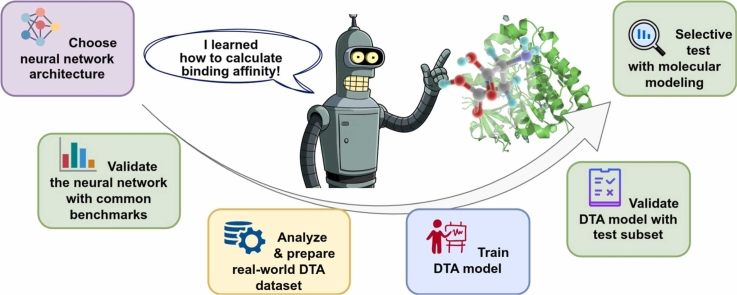

## 1 Introduction

The development of a new drug that gains marketing approval is estimated to cost USD 2.6 billion, and the approval rate for drugs entering clinical development is less than 12 % [1]. Classical laboratory screening technologies, which measure the affinity of a small molecule to a target protein remain labor-intensive and expensive. Modern advances in computational methods and technologies, including the application of machine learning (ML) to chemical and biological research, make it possible to simultaneously expand the search space and to decrease the number of lead molecules.

One of the important tasks in drug design is predicting binding affinity for protein-ligand complexes (DTA, Drug Target Affinity). Modern computational approaches to DTA prediction, which relate to the category of QSAR (Quantitative Structure–Activity Relationship), are based on various machine learning methods that have been actively developed in recent years, mainly neural network-based. To train a DTA model, it is necessary to have reliable datasets with experimentally measured affinity constants (e.g., Ki, IC50). There are datasets traditionally used as a benchmark: Davis [2] and KIBA [3], but they contain information on only tens of thousands of protein-ligand complexes, which is insufficient for training highly effective models. In contrast, large scale affinity measurement databases containing about a million records of measurements, such as BindingDB [4], PDBbind [5], BindingMOAD [6], are not directly ready for DTA training and strongly need preliminary preparation and filtering.

Predicting drug-target affinity requires machine-readable representation of both proteins and ligands, and the choice of numerical representation for protein and small molecule structures largely determines the success of training a DTA model. Small molecule structure description can be presented as a SMILES [7], a molecular graph, one of the pre-calculated descriptors (ECFP [8], Morgan [9], etc.), or even a 2D-image of the molecule (DeepSnap-DL [10], Image2SMILES [11]). Precalculated descriptors convert a molecular structure into numerical vectors, which entails informational loss. Thus, they are rarely used as a full-volume representation of the molecule. However, some DTA models, like FingerDTA, utilize these descriptors [12]. Besides, the graph form is the most natural representation of a molecule structure. This approach was used in the development of the following models: GraphDTA [13], DGraphDTA [14], MGraphDTA [15], DoubleSG-DTA [16], HSGCL-DTA [17]. However, using graph representation leads to a number of complications in neural network architecture. The SMILES notation is ultimately the same molecular graph, expanded as a string and allows applying the neural network approaches from the NLP field (Natural Language Processing), mainly the transformer-based neural networks. As a result, the SMILES-based representation of a small molecule is increasingly used in the field (ProSmith [18], DeepDTA [19], MRBDTA [20], MFR-DTA [21]). Some studies combine several representation methods in one multimodal neural network to gain some advantage in the representation completeness (MultiScaleDTA [22], HGRL-DTA [23], 3DProt-DTA [24], BiComp-DTA [25], MSF-DTA [26], HGTDP-DTA [27]).

Protein encoding methods also vary. For example, the FingerDTA [12] model uses a relatively simple approach - the Word2Vec model [28]. At the same time the MultiScaleDTA [22], HGRL-DTA [23] and 3DProtDTA [24] models use a 3D-structure generated by AlphaFold [29]. In the ProSmith [18] work authors used a primary amino acid sequence as text.

To date, some of the most notable DTA models are ProSmith [18], DeepDTA [19], and HGTDP-DTA [27]. The ProSmith DTA model [18] employs a multimodal Transformer Network to process a protein’s amino acid sequence together with a ligand’s SMILES string as an input pair. This approach estimates interplay between the protein and ligand structures, attempting to predict their structural and therefore functional interaction. Proteins are represented using the ESM-1b [30], and ligands via ChemBERTa-2 [31]. The ProSmith model was pre-trained for six epochs on the BindingDB dataset [4], which contains affinity values for approximately one million protein-ligand pairs. Then the obtained neural network parameters were used to continue training this model on the Davis benchmark dataset [2]. The authors used this approach due to the insufficient amount of data in the Davis dataset for training (∼30,000 records). At the time of publication, the ProSmith model performed the best at the Davis benchmark. An alternative approach for representation of proteins and small molecules is used in the DeepDTA model [19] and based on the integer encoding for both the protein and the ligand (e.g., C:1, H:2, N:3, etc.) in a convolutional neural network (CNN) with two separate inputs. The HGTDP-DTA affinity model [27] processes a ligand, represented as SMILES, and a protein as the amino acid sequence and then transforms it into molecular graphs to obtain numerical vector representations (embeddings) with a GCN. Both molecular graphs were combined into a single feature space using GCN and Transformer.

In this work we aimed to create a new efficient and accurate deep learning model for predicting drug-target affinity. We developed a new approach, called DrugForm-DTA, which uses only the primary amino acid sequence and the SMILES as inputs. Our model uses a relatively simple neural network architecture based on Transformer, shifting the focus from the complexity of the neural network structure to the quality of the model training procedure and the training dataset. The DrugForm-DTA model was tested on standard benchmarks - Davis and KIBA - and compared with state-of-the-art DTA methods for the year 2024: MultiscaleDTA [22], HGRL-DTA [23], MFR-DTA [21]), DGraphDTA [14], BiComp-DTA [25], MSF-DTA [26], GraphDTA [13], DoubleSG-DTA [16], 3DProtDTA [24], HGTDP-DTA [27], MGraphDTA [15], HSGCL-DTA [17], MRBDTA [20], FingerDTA [12]. In order to obtain a highly accurate DTA model, which is applicable for real-world usage, we prepared a large and thoroughly processed training dataset based on the BindingDB database. BindingDB is one of the largest public databases and contains more than 2.8 million experimental measurements of affinity constants. We provided the prepared training dataset along with its test part, separated using a combination of cold target and drug scaffold splits. We trained a DTA model on the prepared BindingDB dataset and demonstrated high efficacy in predicting the affinity constants of a small molecule for a protein. The DrugForm-DTA model has an advantage compared to approaches that require the presence of the 3D protein structure.

## 2 Materials and methods

### 2.1 DTA model training

Training a DTA model requires a neural network architecture and a prepared dataset. Neural network accepts molecules represented as numerical tensors, so ligand and protein encoders are required as a connection link between the neural network and the dataset. The training dataset includes a thoughtful preparation as well as a train-test split procedure.

#### 2.1.1 SMILES

The SMILES (Simplified Molecular Line Entry System) notation is a string representation of a molecular graph [7], both machine- and human-readable. SMILES has become one of the standards for representing the molecular structure in chemoinformatics. On the one hand, SMILES is easy to convert into molecular structure, unlike the human-readable IUPAC (International Union of Pure and Applied Chemistry) name [32]. The possibility of converting SMILES into a traditional IUPAC name has also been shown [33]. On the other hand, unlike the machine-readable InChI (International Chemical Identifier) notation [34], SMILES explicitly represents a molecular graph, and small changes in the structure lead to small changes in the SMILES notation, which makes SMILES an optimal option for representing a molecule in machine learning approaches.

#### 2.1.2 Transformer

Transformer-based neural networks are widely used in various ML tasks, allowing to achieve outstanding results in different areas of computer science: natural language processing (NLP), computer vision (CV), etc. The original Transformer is presented by Vaswani et al. [35] as a tool for solving the machine translation task (Seq2Seq). The main idea is to overcome the main problem of recurrent networks - the vanishing gradient problem. Thanks to the trained attention mechanism, the model can possess complete information about the entire data sequence at any moment. Self-attention identifies which elements of the input sequence are relevant in relation to its other elements. Decoder-encoder attention identifies which elements of the input sequence are relevant for inference of the next element in the output sequence.

The original Transformer was used to develop the BERT (Bidirectional Encoder Representations from Transformers) [36] and GPT (Generative Pretrained Transformer) [37] architectures, various variations of which are used by researchers in most modern machine learning tasks.

#### 2.1.3 Chemformer

We use Chemformer to encode ligand molecules into embeddings [38]. It is a transformer-based model based on BART (Bidirectional Auto-Regressive Transformer) [39], a combination of BERT and a transformer-decoder. The Chemformer model is pre-trained on a large set (100 million) of molecules represented as SMILES. The “combined” variant was used, trained with masking in combination with SMILES augmentation, meaning Chemformer is not sensitive to SMILES representation changes. A special experiment is presented in the Supplementary section “SMILES robustness issues”. The embedding dimension size is 512.

#### 2.1.4 ESM-2

To encode proteins into embeddings, we use the ESM-2 model (variant ESM2_T30_150M_UR50D) [30]. This 30-layer transformer-based model was trained on ∼27 M amino acid sequences and is primarily designed to predict 3D protein structures from a given sequence. The language model underlying ESM-2, unlike another well-known protein structure prediction model, AlphaFold [29], has comparable accuracy but requires less computation time. In addition, AlphaFold2 and other alternative models use the multiple sequence alignment (MSA) to achieve optimal performance. However, ESM-2 generates structure predictions using internal representations of the language model and requires only the primary amino acid sequence as input. The embedding size is 640.

#### 2.1.5 Training procedure

The training data contains sparse target values: both pKi and pIC50 values are not always available, but only one of them. Multitask training successfully deals with missed target values (NaN): training loss function is modified in order not to affect those network weights which are not connected to the missing output target. Since the pKi and pIC50 values correlate, training one of the parameters improves the prediction of the second parameter.

The prepared dataset was divided into training and test subsets, and the test subset did not participate in the training procedure in any role. A combination of cold target split and drug scaffold split was used in order to overcome weaknesses of naive random split in the DTA task. The subject of splits in DTA is described in the “Training-test split issues” section in the Supplementary, where different splits are explained and benchmarked.

Cross-validation training is used to prevent any risk of overfitting and to obtain a robust model. The training subset was divided into training and validation subsets in four parts without crossing the validation sets (classical K-fold CV [40]). Each submodel learned on the 3/4 of the full training set, and validated at 1/4 of the full training set. The validation subset is used as overfitting control and model selection criterion. This approach improves the predictive power of the model at the cost of additional computations.

The neural networks used in the model are computationally heavy, but the embeddings produced by the non-trainable ligand and protein encoders do not change over time and were cached. Thus, the additional cost of introducing four submodels is less than four times. At inference step outputs of all submodels are averaged.

Thus, four submodels were trained on their own CV splits. It took 30 days to train each submodel at NVidia A100 GPU. Architecture and training parameters were identical for each submodel, including: 6 decoder layers, 1 encoder layer, ReLU activation, RAdam optimization procedure with learning rate 0.0001, batch size 64 and early stopping with up to 60 epochs. Full details on the architecture and training procedure are available in the training code (see Data Availability). Each of the four submodels contain 31.8 M trainable parameters.

All program code is written in Python using the PyTorch framework version 1.13. The implementation of transformer layers is taken from the fairseq library [41]. We also used RDKit v2023.9.5 and MolVS v0.1.1. to process and standardize SMILES.

#### 2.1.6 Benchmark metrics

MSE (Mean Squared Error) is a basic regression metric, used in benchmarks. RMSE (Root Mean Squared Error) is calculated as a square root of MSE. Both metrics could be obtained from each other. Still, RMSE has a remarkable feature to reflect the distance from the target values, because they have the same scale. Thus, we used RMSE to estimate performance of the trained model.

C-Index (Concordance Index) is a measure of the rank correlation between the predicted risk values and the observed time points [42]. Originally it is a classification metric, but it could be adapted to regression tasks. To calculate the metric values, we used the code from the Affinity2Vec work [43]. We followed the established tradition in DTA works, but we see this metric as a questionable choice for a pure regression task.

In this work we compare our benchmark results with the numbers, given in the HGTDP-DTA [27] work, where authors collected and organized into a table the Davis and KIBA benchmark tests on a large number of other DTA models.

#### 2.1.7 Confidence interval

This paper uses confidence interval estimations in several cases using both the Student criterion and the bootstrap algorithm. The first method involves coefficients from the t-distribution table: $\mathrm{CI}=\;t(N)\frac{\sigma }{\sqrt{n}}$. The bootstrap algorithm estimates the distribution of an estimator by resampling (often with replacement) one’s data or a model estimated from the data. Its advantage is not requiring assumptions about the data distribution. In this paper, the confidence interval of the experimental affinity constants values is calculated in both ways with close results. In addition, we can estimate the confidence interval of the trained affinity model forecasts. Confidence interval of estimation can be calculated as CI = z∙SE. With assumption of normal sampling distribution standard error of estimation (SE) is numerically equal to RMSE for large N. The value of the reliability parameter of the confidence interval is the same in all cases: 95 % (2σ), the value of corresponding the z-score t = 1.96. See Figure S4 in the Supplementary.

### 2.2 DTA benchmark datasets

To evaluate the ability of the DrugForm-DTA network to solve drug-target affinity tasks, the commonly used Davis and KIBA datasets were used.

#### 2.2.1 Davis

The Davis dataset is a regression dataset that includes 68 drugs and 279 unique protein kinases [2]. In total, Davis contains quantitative Kd values for 25,772 ligand-protein pairs, which are calculated using the formula:

$$pK_{d}={-\log}_{10}(K_{d}\times 10^{-9})\;$$

This dataset is traditionally used as the main benchmark in DTA tasks. The dataset was preliminarily divided into a training and a test set, according to the instructions of the benchmark authors. Drug molecules are presented as SMILES, protein amino acid sequences are obtained from the UniProt database [44]. The Davis dataset values distribution is presented at Figure S5.

#### 2.2.2 KIBA

The KIBA (Kinase Inhibitor Bioactivity) dataset is a regression dataset that includes 2068 ligands and 229 unique protein kinases [3]. In total, KIBA contains quantitative binding values for 117,657 ligand-protein pairs.

A distinctive feature of the dataset is the unification of different affinity values (Ki, IC50) into a common entity - KIBA score, which is a modified Ki:

$$K_{i,\mathrm{adj}}=\;\frac{{\mathrm{IC}}_{50}}{1+0.3({\mathrm{IC}}_{50}/K_{i})}$$

This dataset is one of the standard ones traditionally used as a DTA benchmark. The dataset was preliminarily divided into a training and a test set. The drug molecules are presented as SMILES, the protein amino acid sequences were obtained from the UniProt database [44].

### 2.3 BindingDB

The DrugForm-DTA model was trained for practical application using the BindingDB database.

#### 2.3.1 Dataset description

BindingDB is a curated dataset launched in 2000 and updated weekly [4]. It collects affinity constant values from papers, patents, and other sources, including user-uploaded ones. Among other large datasets such as PDBBind [5] or BindingMOAD [6], BindingDB is the largest, containing 2875,634 protein-ligand pairs (1238,443 ligands and 6523 proteins), version “ALL_202406”.

#### 2.3.2 Dataset preparation

The downside of the large size of the BindingDB dataset is the significant amount of invalid data and records not related to human organism. In addition, the dataset often has more than one measurement available for a single protein-ligand complex, making it impossible to train a model without prior data adaptation. We used a complex filtering procedure to prepare the dataset for training. We inserted missing UniProt IDs and obtained full amino acid sequences, then applied mutations, given in the BindingDB record, to the sequences obtained from UniProt [44]. We filtered the records: without affinity values, from inappropriate organisms, obtained at non-physiological temperature and acidity values, with invalid SMILES, with too large and too small proteins.

Regarding organism filtration, proteins of bacteria and their viruses were removed, since conditions (like temperature, pH) and the structure of their cytoplasmic membrane and cell wall differ significantly [45], [46]. We wanted to work more carefully and analyze proteins like biological entities. When filtering proteins by the weight, records with less than 50 amino acids were excluded, since they are peptides, as well as more than 2500 amino acids, since we tried to avoid huge polypeptide complexes such as ribosomes. For the same reason, a filter by weight from 1 to 500 kDa was applied.

**Fig. 1:**
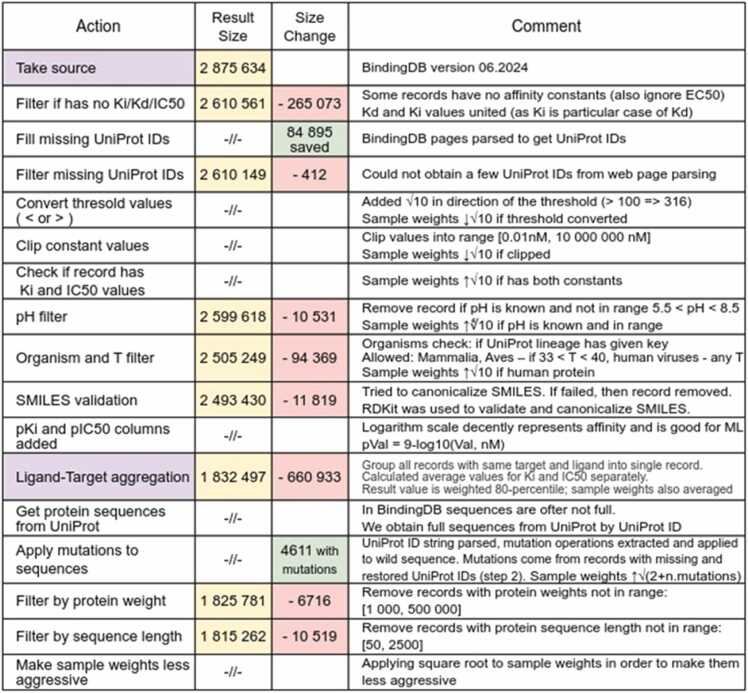
The BindingDB dataset preparation pipeline.

We also clipped values outside the relevant range (out of 99th percentile) and normalized the values to log scale. In addition, we performed an aggregation procedure to unite values for identical protein-ligand complexes. Each pair was weighted according to the degree of confidence in the value and the value of the example. A complete representation of the filtering procedure is presented in Fig. 1, indicating how many records does each operation reduce the dataset.

Here we describe some of these steps. For ∼85,000 records from the BindingDB database, the UniProt ID of the protein is not specified, so the link to the UniProt ID was extracted from the BindingDB website [4]. The UniProt IDs were restored for 4611 unique protein-ligand complexes, including the mutant proteins.

The BindingDB dataset contains the Kd, Ki and IC50 affinity constants separately. We combined the constants Kd and Ki into a common column, but left IC50 as a separate target. We can consider Ki as a special case of Kd, but the Ki and IC50 constants, although close numerically, have different nature, so we cannot unite them into a single entity [47].

We transformed threshold values (for example, >5, <6) into exact ones based on a 10x dilution hypothesis [47]. To convert the threshold value into the exact one we shift it by half of the dilution step towards the threshold side, meaning multiplying or dividing by √10 ≈ 3.16. For example, the threshold value Ki≥ 10^4^ is converted to the exact value 3.16·10^4^, while simultaneously reducing the weight (importance) of this sample in training. Justification of this procedure is given in the Supplementary section “Binding affinity experimental measurement issues”. Thus, instead of ∼75,000 records with pIC50= 6 in the final dataset, we get ∼37,000 of pIC50 = 6, ∼19,000 of pIC50 = 5.5 and ∼19,000 of pIC50 = 6.5. For the sake of training stability and analysis simplicity, we convert them to a logarithmic scale: $\mathrm{pKi}=9-\log_{10}(\mathrm{Ki},\mathrm{nM})$, $\mathrm{pIC}50=9-\log_{10}(\mathrm{IC}50,\mathrm{nM})$.

If multiple records for one protein-ligand pair are available, we aggregate them into a single record with pKi and pIC50 values calculated as 80th percentile of all available measurements [48]. Three hundred randomly selected examples of visualizing 80th percentile calculation can be downloaded by the link given in the Data Availability section. These figures also contain the value spreads and the confidence intervals of the experimental measurement (95 %).

Thus, along with filtering and transforming the affinity values, we obtained their weights (significance of each training sample). Initially, all weights were equal to “1”. During dataset processing, the record weight was multiplied by a certain factor if it is important, or divided by it if the significance of the record is questionable. A typical value of the factor for a significant criterion is √10 ≈ 3.16, i.e., 1/2 order, and ∜10 ≈ 1.8 (1/4 order) for an insignificant criterion. A special case is increasing the importance of records with mutations. The more mutations are in the protein, the more valuable this example is. Therefore, the increasing coefficient for them is

$\sqrt{\left(2+n_{\mathrm{mut}}\right)}$, where $n_{\mathrm{mut}}$ is the number of mutations in the protein. We analyzed the resulting distribution of weights and decided that it is too strong (the range of values is from 0.1 to 10), so we lightened it out using the square root operation and got the range of weights from 0.3 to 3.

#### 2.3.3 BindingDB selective subset

To conduct detailed testing of the DTA model, we manually selected a small subset of protein-ligand complexes from samples not involved in model training. This set contains 7 proteins: melatonin receptor 1 A (MTR1A), melatonin receptor 1B (MTR1B), orexin/hypocretin receptors type 1 (OX1R) and type 2 (OX2R), blood coagulation factor XIa (FA11), epidermal growth factor receptor (EGFR), sodium- and chloride-dependent glycine transporter 1 (SC6A9). These proteins are targets for the creation of antitumor drugs, drugs for insomnia and depression, as well as anticoagulants. We chose the ligands for them from the BindingDB test set (scaffold split) with affinity values in a wide range for positive and negative control. As a result, 7 ligands were selected for the MTR1B protein, and 20 ligands for the other proteins. Thus, the obtained selective subset includes 127 protein-ligand complexes.

### 2.4 Molecular modeling methods

We performed several case studies, comparing our model performance with the ability of molecular modeling methods to estimate binding scores, including molecular docking, calculation of binding energy using molecular mechanics and semi-empirical quantum chemistry evaluation. The testing was performed at 127 protein-ligand complexes from the selective subset.

#### 2.4.1 Molecular docking

We have chosen crystal structures of the proteins in the selective subset from the RCSB Protein Data Bank (PDB ID: 4ZJ8, 6TPN, 8A27, 4CRC, 6ME2, 6ZBV, 6ME6) [49]. All structures were obtained by X-ray diffraction, structures with good resolution (<2 Å) and no missing atoms and amino acid residues were a priority. Molecular docking and pre-preparation of proteins and ligands were performed using Schrödinger Suite [50]. Protein structures were prepared using Protein Preparation Wizard [51], and ligand structures were prepared using the LigPrep tool [52] taking into account stereoisomers and ring conformations. Proteins and ligands were parameterized in the OPLS force field [53]. Molecular docking was performed using the Glide docking program [54]. A potential grid with the size of 20 × 20 × 20 Å was pre-built for each receptor. Glide performs a complete systematic search of the conformational, orientational and positional space of the ligand in the protein active site. In this search, an initial coarse positioning and scoring phase, which dramatically narrows the search space, is followed by torsional flexible optimization of energy on an OPLS-AA non-bonded potential grid for a few hundred surviving candidate poses. The selection of the best docked ligand pose uses a model energy function that combines empirical and force field-based values. Molecular docking was performed for each ligand conformer in XP (extra precision) mode, and the ligand with the best Glide score values was used for subsequent modeling steps.

#### 2.4.2 Molecular mechanics

We calculated the free energy of protein-ligand binding (∆G_*bind*_) using molecular mechanics algorithms. The calculations were performed by molecular mechanics methods using the generalized Born model MM-GBSA (General Born Surface Area) [55] implemented in the Prime Schrödinger software package [56]. To calculate the binding energy of MM-GBSA, the position of the ligand in the protein active site obtained as a result of Glide docking was used. The binding energy ∆G_*bind*_ was calculated using the formula:

$$∆G_{\mathrm{bind}}\;=\;∆G_{\mathrm{solve}}-∆E_{\mathrm{MM}}-∆G_{\mathrm{SA}},$$

where ∆G_*solve*_ — is the difference in the solvation energy of the GBSA protein-ligand complex and the sum of the free protein and free ligand solvation energies, ∆E_*MM*_ - is the difference in minimized energies between the protein-ligand complex and the sum of the free protein and free ligand energies, ∆G_*SA*_ - is the difference in the surface area energies of the complex and the sum of the free protein and free ligand surface area energies. The method also allows the calculation of the ligand deformation energy by placing the ligand in solution using the VSGB 2.0 solvation model. In addition, the MM-GBSA energy calculation allows the estimation of individual energy contributions such as electrostatic interaction energies, van der Waals interaction energies, lipophilicity and covalent interaction energies, as well as corrections for hydrogen bonds, *π−π* stacking, to the total binding energy.

MM-GBSA binding free energy calculations have several advantages. They are more theoretically rigorous than the empirical estimates used in molecular docking, and at the same time less computationally expensive than binding free energy modeling.

#### 2.4.3 Quantum chemistry methods

We used semi-empirical quantum chemistry methods to more accurately predict the protein-ligand binding affinity for the selective subset. Quantum chemistry methods allow eliminating false positive results that occur when using molecular docking. The protein-ligand binding enthalpy was calculated by the PM7 method [57] using the ligand position in the active site of the protein obtained as a result of molecular docking. In the first step, the energy of the protein-ligand complex was locally optimized using the PM7 method. During optimization, the coordinates of all ligand atoms were varied, and all protein atoms were fixed. Then, the energy of the complex was recalculated using PM7 and the COSMO solvent model [58] for all fixed atoms (1SCF+COSMO). The energy of unbound ligand formation is calculated similarly - local optimization of the energy of macrocyclic and non-aromatic ring conformations generated by LigPrep [52] and subsequent optimization taking into account the solvent (1SCF+COSMO) were performed. Among all ligand conformations, the enthalpy with the minimum value was chosen as the enthalpy of unbound ligand formation. The energy of the unbound protein was calculated by the 1SCF+COSMO method for a fixed protein conformation, which was used for molecular docking.

The enthalpy of binding was calculated using the equation:

$$∆H_{\mathrm{bind}}\;=\;∆H_{\mathrm{complex}}-∆H_{\mathrm{protein}}-∆H_{\mathrm{ligand}},$$

where ∆H_*complex*_ is the enthalpy of optimized complex formation, ∆H_*protein*_ is the enthalpy of unbound protein formation, ∆H_*ligand*_ is the enthalpy of unbound ligand formation. The calculated enthalpy values were used to rank the affinity of the ligand to the protein.

#### 2.4.4 Modeling score

We developed an algorithm to combine the results of ligand-protein binding measurements for the three approaches: molecular docking, MM-GBSA analysis, quantum chemistry methods (PM7 +COSMO). This algorithm aggregates all molecular modeling methods into a single score which is required because each method evaluates protein-ligand binding in different quantities: docking score, MM-GBSA binding energy (∆G_*bind*_) and quantum chemistry binding enthalpy (∆H_*bind*_).

To calculate a single modeling score, a preliminary ranking procedure was performed in order to assign a category for each value. The ranges of Glide docking scores were: [−20;−7) → high, [−7;−5) → medium, [−5;0) → low. For molecular mechanics, the ranges were: [−150;−70) → high, [−70;−50) → medium, [−50;0) → low. And the quantum chemistry approach ranges were [−100;−50) → high, [−50;−30) → medium, [−30;0) → low. All other values outside the ranges are assigned the category modeling failed. Next, the category is converted into a numeric value: high → 1, medium → 0.5, low → 0, failed → −1.

The final modeling score was the arithmetic mean of these scores.

$$\mathrm{Modeling score}=\;\frac{1}{N}\sum_{i=1}^{N}\mathrm{Rank}k_{i},$$

where *N* - a number of molecular modeling methods, *Rank k*_*i*_ - the value of the ranks of the *i* method.

## 3 Results

### 3.1 DTA neural network architecture

**Fig. 2:**
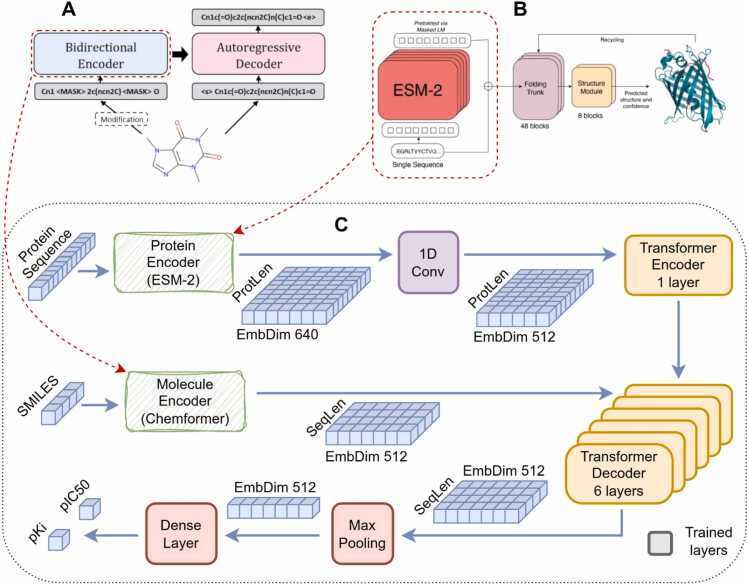
Proposed neural network for DTA prediction. A: Using the Chemformer model (combined variant) as a non-trainable molecule embedding generator. B: Using ESM-2 model (ESM2_t30_150M_UR50D variant) as non-trainable protein embedding generator. C: DrugForm-DTA neural network architecture. The decoder block takes the molecular embedding as input, while conditioning with protein embeddings through the encoder block. The 1D-convolution layer is fixing the difference between molecule and protein embedding dimensions.

Neural networks are one of the most advanced approaches to solving QSAR problems. Transformer-based neural networks [35] effectively process string sequences of different lengths. This is particularly advantageous in the DTA problem, as ligand and protein molecules are represented by a SMILES string and an amino acid sequence, respectively. Here we extensively used neural network architecture for solving the DTA problem, taking into account recent advances in the field [18], [30], [38]. We tested two neural network structures. The first one is a BERT-like network (encoder-only), in which the input sequence is formed by concatenating ligand and protein embeddings with a separator. This network is similar to ProSmith [18], but uses a different molecule encoder - Chemformer [38] (combined variant, Fig. 2A), which we consider more promising than ChemBERTa-2 [31]. Chemformer performs significantly better than ChemBERTa-2 on the MolNet benchmark [59].

(a) Adopted from [38]. (b) Adopted from [30].

The ESM-2 model (ESM2_t30_150M_UR50D, Fig. 2B) is used to generate protein embeddings [30], and the ligand and protein embeddings must have the same dimension for concatenation. The ligand embedding has a dimension of 512, and the protein embedding has a dimension of 640, so it must be converted to the same dimension. To this end, we applied a 1Dconvolution which performs the same function instead of a linear layer as in the ProSmith work [18].

A minor disadvantage of our initial network structure is the increased size of the encoder input (protein & molecule) due to self-attention to the *O(N*^*2*^*)* complexity. The proposed network structure is a vanilla transformer consisting of an encoder and a decoder (Fig. 2C). The protein embedding is fed to the encoder and determines the decoder’s behavior through encoder-decoder attention. The final network requires fewer calculations than the initial one, since ${\mathrm{len}}_{\mathrm{prot}+\mathrm{mol}}^{2}>{\mathrm{len}}_{\mathrm{prot}}^{2}+{\mathrm{len}}_{\mathrm{mol}}^{2}$.

Thus, the neural network accepts a SMILES string and a primary amino acid sequence as inputs. The SMILES string is processed by the Chemformer encoder, and the protein sequence is processed by the ESM-2 encoder. The Transformer decoder block contains 6 layers, and the Transformer encoder block contains only one layer, as features are already extracted by ESM-2. A Max Pooling layer in combination with a Dense layer converts the decoder embeddings into a vector of output values, representing the affinity between a given ligand and protein.

### 3.2 DTA benchmarks

The proposed neural network architecture was tested on the traditionally used Davis [2] and KIBA [3] benchmarks. The KIBA and Davis datasets require a regression approach for model evaluation, so the primary benchmarking metric is MSE (Mean Squared Error). We used the RMSE (Root Mean Squared Error) metric, because it numerically corresponds to the range of target values. To compare DrugForm-DTA with other models, the values were converted back to MSE. In addition to MSE we also used the C-index to compare performances across models.

**Fig. 3:**
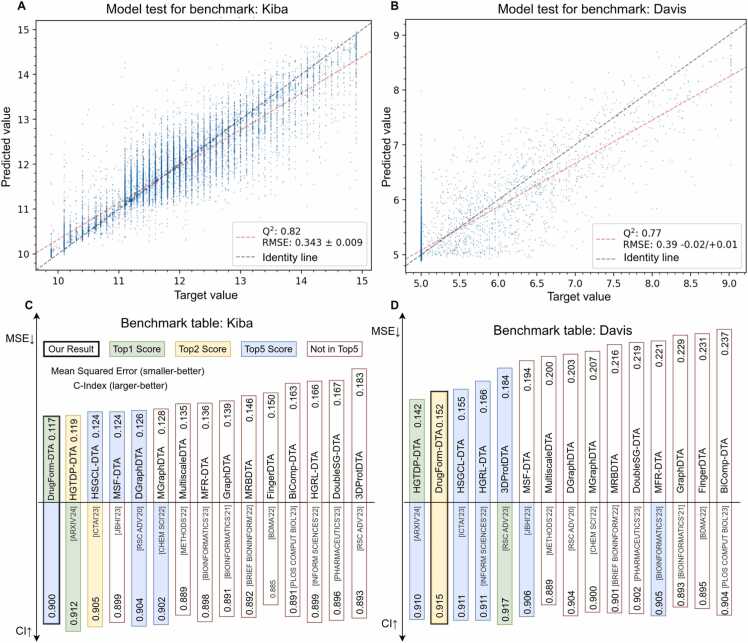
Performance of the DrugForm-DTA model on the Davis and KIBA datasets. A: Visualization of the prediction on the KIBA test subset. There is a moderate correlation between the model predictions and the target values. The dataset is dominated by rounded values. B: Visualization of the prediction on the Davis test subset. The dataset contains few values except 5.0, so the correlation is weaker than for KIBA. C: Comparison of prediction performance across DTA models on KIBA benchmark. DrugForm-DTA ranked first in MSE metric with a value of 0.117. D: Comparison of prediction performance across DTA models on Davis benchmark. DrugForm-DTA ranked second with MSE= 0.152.

We trained separate models on the Davis and KIBA benchmark datasets. At this stage BindingDB was not used in any way. First, we evaluated the correspondence between the trained model predictions and target values on the test subsets. For both the KIBA (Fig. 3A) and Davis (Fig. 3B) benchmarks our results indicate a moderate correlation between the model predictions and the target values. Moreover, the points clustered on the left side of the plot (Fig. 3B) correspond to records with the target value pKi= 5, which dominate in the Davis dataset. This observation highlights a limitation of benchmarking datasets that requires further investigations to enable proper interpretation of predictions and reliable model evaluation.

Next, we compared the DrugForm-DTA model’s performance with that of other published DTA models. Despite the previously mentioned issues in the Davis dataset, we benchmarked DrugForm-DTA against other methods using the same data to evaluate model performance. The DrugForm-DTA model achieved the top-1 performance on the KIBA benchmark (Fig. 3C) (MSE=0.117, C-index=0.912), and on the Davis benchmark (MSE=0.152, C-index=0.915) our model was the second best (Fig. 3D). We also evaluated the C-index metric, and DrugForm-DTA outperformed HGTDP-DTA. However, the 3DProt-DTA approach achieved the best C-index metric across all models but was significantly inferior in MSE (MSE=0.184).

Overall, we benchmarked the DrugForm-DTA model against other approaches and observed high performance of our method. We used both MSE and C-index metrics, which can be discordant in their ranking across methods. We also highlight limitations in the benchmarking datasets that require further investigations to enable proper interpretation of model performance.

### 3.3 BindingDB preparation

Building an efficient and accurate affinity model requires a high-quality, consistent dataset. So, we performed additional steps to prepare the datasets and train the model using the most reliable instances. We obtained binding affinity values from the public BindingDB database [4] to train DrugForm-DTA, which contains more than 2.8 million experimental measurements (1238,443 ligands and 6523 proteins). However, we identified several issues that degrade the quality of the training data: the presence of threshold values, multiple measurements for the same protein-ligand complexes, incomplete protein annotations. We defined an approach to address these issues.

**Fig. 4:**
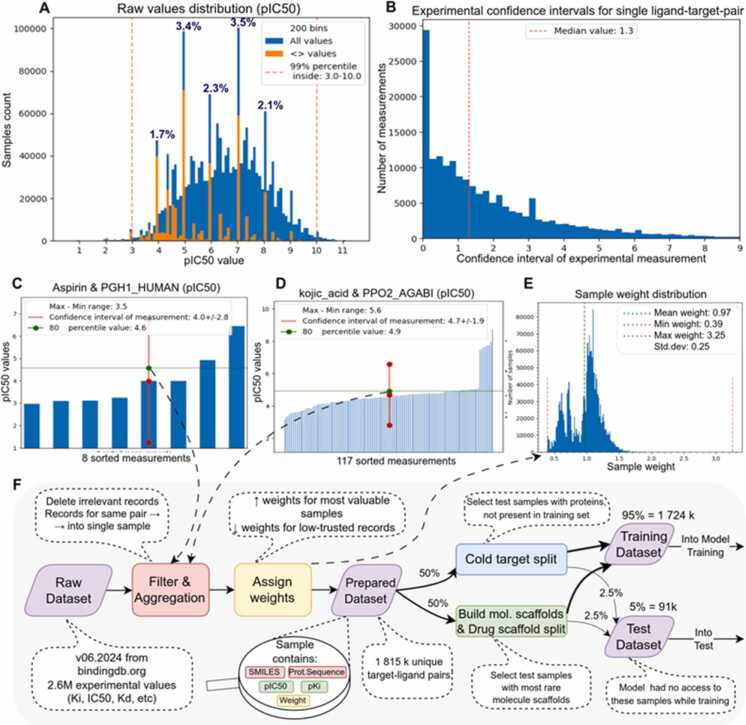
Preparation of the BindingDB dataset. A: Distribution of raw values in the dataset (using pIC50 as an example). Orange color shows how many values are specified with the < or > threshold. Their occurrences in percentages of the whole dataset are indicated above the peaks. B: Histogram of the confidence interval values of the experimental measurement of affinity constants for individual protein-ligand pairs. Zero values are for pairs with identical values. Even taking them into account, the median value is 1.3 orders of magnitude. C: An example of aggregating several measurements for one protein-ligand pair: the 80th percentile represents a soft maximum that is robust to outliers and will be used in training. D: An example of robustness to outliers: a group of measurements with results significantly exceeding the norm is discarded. 80th percentile value (4.9) is close to average value (4.7) and quite far from maximal value (8.7) E: Distribution of sample weights in the final dataset. Weight value larger for more reliable samples. F: Schematic diagram of the original BindingDB database transformation into training and test datasets.

We observed that many experimentally measured binding affinity values in BindingDB are reported (Fig. 4A) as a threshold values (e.g., >5, >6). We hypothesize that a serial dilution method underlies this pattern. Excluding these records leads to information loss, and simply removing the thresholds leads to significant deviations in accuracy (about half an order of magnitude). As a compromise we replace threshold values with an estimated true value by adjusting the reported threshold affinity value by 1/2 order of magnitude away from the threshold. Justification of this procedure is provided in the Supplementary section “Binding affinity experimental measurement issues”. Moreover, this approach also smooths the distribution of measurements reported as threshold values, which are anomalously overrepresented. Thus, modifying these records prevents the neural network from learning a specific value instead of approximating the hypersurface of the function being optimized [60].

One of the key issues is the presence in BindingDB of multiple different measurements for 43 % of the unique protein-ligand complexes. For each

protein-ligand pair with two or more measurements we estimated the 95 % confidence interval (Fig. 4B) and the difference between the maximum and minimum values (Figure S6). An average confidence interval of 1.3 orders and an average min-max difference of 0.9 orders appear substantial and indicate inconsistency in the experimental data. For example, available records in BindingDB for a fundamental complex such as Aspirin & PGH1_HUMAN span a wide range from 3 to 6.5 (Fig. 4C). Existing approaches [61] for handling multiple measurements suggest using the mean or maximum value. The motivation for taking the maximum value instead of the mean value is that an experimental error is more likely to decrease the affinity than to increase it [48] (see the “Binding affinity experimental measurement issues” chapter in the Supplementary). However, using the strict maximum is not robust to outliers. As a compromise we use the 80-percentile value, which behaves like a soft maximum but robust to outliers.

An example of such robustness to outliers is the aggregation of pIC50 values for the pair kojic acid & PPO2_AGABI (Fig. 4D), where the 80-percentile value is close to the mean and disregards the cluster of high values that deviates from the main distribution. We also note that in practice we take a weighted percentile. The weights (importance) of records are derived from various factors which make an individual record more or less reliable (Fig. 4E).

We observed that the BindingDB dataset sometimes provides only the binding site region instead of the full protein sequence, and occasionally the sequence contains incorrect symbols that do not present in the standard amino acid alphabet. Therefore, the amino acid sequences were obtained directly from the UniProt database [44]. In addition, information of the protein host organism also was extracted from UniProt.

The overall BindingDB filtration and transformation procedure (Fig. 4F) allowed the preservation of as much data as possible without significant dataset contamination. The final dataset contains 1739,873 protein-ligand records (5251 proteins, 1020,614 ligands) with the following parameters: amino acid sequence, SMILES, pKi, pIC50, weight.

The final step in preparing the dataset for model training is splitting it into training and test sets. We assigned 5 % of the dataset as the test set - more than 90,000 records. This amount is comparable to that of full datasets used in some works based on BindingDB (ELECTRA-DTA [62] 144,000 protein-ligand pairs, BiComp-DTA [25] - 42,000, SAM-DTA [63] - 232,000), and is sufficient for training a high-quality DTA model. Random split is considered suboptimal [59], [64], [65], so we constructed half of the test set using a cold target split, and the other half using a drug scaffold split.

Overall, the filtering and transformation of the BindingDB data constitute a key component of our approach to training a high-performance binding affinity model. We also consider this data preparation step one of the most important steps toward developing a model capable of accurately predicting ligand-target affinities. Subsequent steps involve the computational techniques used to train and evaluate the DrugForm-DTA model.

### 3.4 Training and performance of the DrugForm-DTA model

**Fig. 5:**
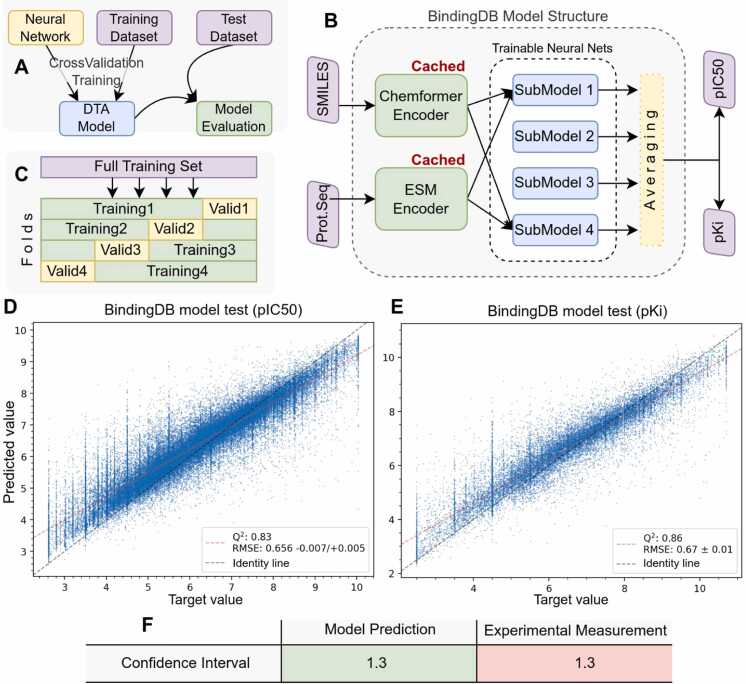
DTA model trained on the BindingDB dataset. A: Splitting the original dataset into training and test subsets. B: Structure of the DTA model. The model consists of several submodels, one for each cross-validation fold. The results of the submodels are averaged to obtain a consolidated value. The protein and ligand encoders are cached and calculated once. The model takes the primary amino acid sequence and SMILES as input and returns two values at once: pKi and pIC50. C: Splitting the training dataset into a group of training and validation subsets by K-fold cross-validation: individual parts of the dataset alternately become training or validation. D, E: Evaluation of the quality of the trained model using the test set. RMSE(pKi)= 0.66, RMSE(pIC50)= 0.67. There is an observable correlation between the model predictions and the target values. F: Comparison of confidence intervals of experimental measurement and model calculation.

After preparing the optimized BindingDB dataset, we trained the DrugForm-DTA model (Fig. 5A) using a multitask approach to simultaneously predict two parameters: pKi and pIC50. We also used the test set from the BindingDB (90,763 records; see BindingDB Preparation) for independent model evaluation. The DrugForm-DTA model uses SMILES and primary amino acid sequence as inputs, and each string is processed by its corresponding encoder (Chemformer or ESM-2) to generate the required embeddings (Fig. 5B). One of the key elements in the training of the full DrugForm-DTA model is the use of cross-validation and averaging of submodel prediction results (Fig. 5C).

The trained model was applied to the test set. The results are presented in scatter plots separately for pIC50 (Fig. 5D) and pKi (Fig. 5E). The accuracy of the predictions on the test set is RMSE(pIC50)= 0.66 and RMSE(pKi)= 0.67. These values are close to each other, so model accuracy can be estimated using the worst one RMSE= 0.67 (MSE=0.45). The average confidence interval for model predictions on a large dataset is estimated as *CI*_*calc*_ = 1.96 · RMSE = 1.3 (Fig. 5F). We also calculated the confidence interval for a single experimental affinity measurement from the data preparation step: *CI*_*exp*_ = 1.3 (Fig. 4B). Thus, the accuracy of the model predictions is comparable to that of a single experimental measurement. For further details on confidence interval calculation, see the corresponding sections in the Materials and methods and in the Supplementary.

### 3.5 DTA model selective test

#### 3.5.1 Test samples selection

Next, we performed additional computational analyses using molecular docking, MM-GBSA calculations and semi-empirical methods of quantum chemistry to assess the agreement between experimental values and affinity predictions from the DrugForm-DTA and molecular modeling. To this end, we selected entries from the BindingDB test set that were not used during

model training. All selected records correspond to target proteins with therapeutic relevance, have multiple associated ligands spanning a wide range of affinity values, and have experimentally determined X-ray crystal structures available in the PDB [49].

The selected cases include targets for the insomnia treatment: melatonin receptor 1 A (MTR1A), melatonin receptor 1B (MTR1B), orexin/hypocretin receptor type 1 (OX1R), orexin receptor type 2 (OX2R). The crystallographic structures of these target proteins have low resolution (2.6–2.8 Å) and contain missing amino acid residues but these are located away the active site. We also selected ligands with antitumor activity targeting the epidermal growth factor receptor (EGFR). A high resolution (1–1.5 Å) crystallographic structure of the EGFR is currently available in the PDB database, but it contains a significant number of missing amino acid residues. Another example that we considered is blood coagulation factor XIa (FA11) that is used as a target for anticoagulant development. Although the structure has a high resolution (1.6 Å), the amino acid sequence is represented only by the heavy chain and a small region of the light chain. The sodium- and chloride-dependent glycine transporter 1 (SC6A9) was selected as a target for the schizophrenia treatment, the protein’s crystallographic structure has a low resolution (3.4 Å) and approximately 90 amino acid residues missing at the N-terminus of the chain.

Overall, we selected 20 ligands for each protein with a range of affinity values from 2 to 10, except for MTR1B which has only 7 available ligands in the test set. We included this receptor to analyze the model’s behavior for proteins belonging to the same family. Our final evaluation dataset contains 127 protein-ligand pairs and was used to assess the agreement between DTA model predictions and binding energies obtained from molecular modeling methods.

#### 3.5.2 Selective test metrics

**Fig. 6:**
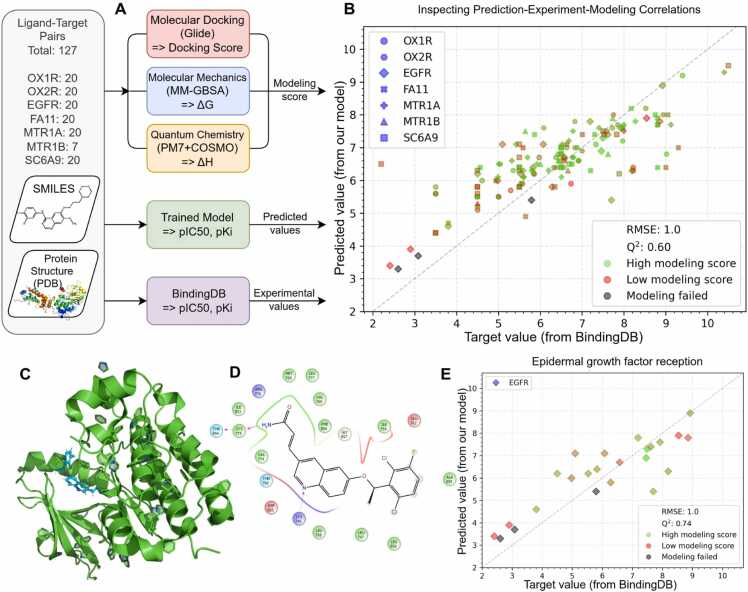
Molecular modeling. A: Flow chart of the detailed testing of single protein-ligand pairs. The modeling score was calculated as the average of the ranked protein-ligand interaction energies obtained by molecular docking (Glide), molecular mechanics (MM-GBSA), and quantum chemical calculations (PM7 +COSMO). B: Scatter plot of the affinities predicted by the DTA model, experimentally measured affinities, and predicted by molecular modeling for 127 protein-ligand complexes. The X-axis shows the experimental affinities, the Y-axis shows the DrugForm-DTA predicted affinities, and the color shows the calculated modeling scores. C: 3D structure of EGFR in complex with the ligand, the ligand position is obtained as a result of molecular docking. D: Position of the ligand in the active site of EGFR. Hydrogen bonds formed with amino acids of the protein are shown with pink dashed arrows. E: Scatter plot of affinities predicted by DTA model, experimentally measured and predicted by molecular modeling methods for EGFR and 20 ligands. All protein-ligand pairs with modeling failed (marked in gray) refer to the EGFR protein.

Next, we applied molecular docking, molecular mechanics and quantum chemical calculations to each protein-ligand complex. We developed an algorithm to combine the results of ligand-protein binding measurements from the three approaches (Fig. 6A).

We performed molecular docking for 127 protein-ligand pairs using the Glide software [54]. Atomistic protein models were suitably prepared based on the crystal structures from the Protein Data Bank (PDB) [49]. Three-dimensional structures of ligands were constructed taking into account the isomerism and charge states, and to approximate the protein-ligand interaction energy, we used the Glide docking score. Additionally, for all protein-ligand pairs we applied MM-GBSA analysis (Prime) to calculate ligand-binding (∆G_*bind*_) affinities [55], using the ligand pose from Glide docking. To obtain the most accurate ligand positioning, we applied quantum chemical methods (PM7 +COSMO) [57], [58] which optimized ligand placement in the protein active site and allowed a more accurate estimation of the binding enthalpies. Finally, we introduced a modeling score representing the mean of the MM-GBSA, Glide and quantum chemistry predicted binding values (see Materials and methods). The obtained modeling scores are continuous, but we split them into classes of low, medium and high modeling scores, and a category where one or more molecular modeling approaches failed to yield a result.

#### 3.5.3 Selective test results

We have compared the affinities from BindingDB with DTA model predictions, and the molecular modeling score (Fig. 6B). We report a low correlation between the modeling score and the experimental affinity values with a Spearman correlation coefficient *C*_*Mod*_= 0.20, whereas DTA model predictions show a significantly higher Spearman correlation coefficient *C*_*DTA*_= 0.76. In particular, protein-ligand complexes with low modeling scores often exhibit high binding affinity in experimental measurements (see the Data availability section).

For the EGFR case the correlation between modeling scores and experimental affinity is also low (*C*_*Mod*_(EGFR)= 0.18), whereas the DTA model prediction shows a high correlation (*C*_*DTA*_(EGFR)= 0.80). As an example, the positioning of a low-modeling score ligand in the EGFR active site was analyzed (Fig. 6C). Glide docking results (Fig. 6D) indicate that the ligand forms three hydrogen bonds with the protein, but the docking score and other modeling methods outputs were low. On the scatter plot (Fig. 6E) we observed a cluster of high modeling scores in the high affinity region. All protein-ligand complexes for which molecular modeling failed belong to the EGFR protein (Figure S7) due to the fragmented PDB structure (the protein is partially crystallized and more than 70 % of the amino acid sequence is missing). Our results illustrate the limitations of the molecular modeling methods for binding affinity prediction when a complete, high-resolution crystal structure of the target protein is unavailable.

We performed a detailed comparison of the molecular modeling results with the experimental and predicted affinities for each of the tested proteins. In particular, modeling of MTR1A (Figure S8) and OX2R (Figure S9) binding revealed high modeling score values for nearly all ligands in the test set, including those with lower experimental values, that leading to low correlation coefficients (*C*_*Mod*_(MTR1A)= -0.03, *C*_*DTA*_(MTR1A)= 0.80, *C*_*Mod*_(OX2R)= -0.26, *C*_*DTA*_(OX2R)= 0.70). Thus, molecular scores are poorly suited to distinguish between high and medium affinities (pKi/pIC50 in the range from 5 to 8). Modeling of ligands binding to OX1R (Figure S10, *C*_*Mod*_(OX1R)= -0.04, *C*_*DTA*_(OX1R)= 0.84) also shows no correlation with experimental data and highlights the need not only for a sufficiently

complete structure - i.e., one containing all amino acid residues - but also need for high resolution (<1.5 Å). Both receptors have missing amino acid residues only at the end of the chain, far from the active site, but their structures have a low resolution (≈2.8 Å). For FA11 (Figure S11, *C*_*Mod*_(FA11)= 0.06, *C*_*DTA*_(FA11)= 0.55) high-resolution crystal structure is available (≈1.6 Å), but the modeling scores do not fully correlate with experiment, likely due to the absence of a significant part of the light chain sequence.

A large number of ligands with low modeling scores are also observed for protein–ligand complexes with SC6A9 (Figure S12, *C*_*Mod*_(SC6A9)= 0.27, *C*_*DTA*_(SC6A9)= 0.70), which represents one of the best modeling results along with MTR1B (Figure S13, *C*_*Mod*_(MTR1B)= 0.40, *C*_*DTA*_(MTR1B)= 0.54).

## 4 Discussion

The main result of this work is the creation of DrugForm-DTA, a new trained model for predicting the affinity of interaction between a ligand molecule and a target protein, capable of predicting the values of pKi and pIC50 constants for any protein-ligand complex. DrugForm-DTA uses a relatively simple and not overcomplicated neural network architecture based on the Transformer, while the focus is shifted from the complexity of the neural network structure to the quality of the model training procedure and the quality of dataset preparation. We are convinced that it is the quality of the data, and not the complexity of the neural network architecture, that is crucial for a successful machine learning model.

We state that high-quality data is a primary source for training an accurate model. There are approaches to enrich the training datasets with artificial data, obtained through docking or other computational methods [66], [67]. Although such approaches can generate very large datasets, we consider training on real experimental data significantly more valuable. Using large-scale artificial datasets is expected to yield better training metrics, but we expect the behavior of the trained model to be far from reality. In this work we demonstrated that molecular modeling calculations have low correlation with experimental values. Moreover, using artificial protein structures in docking - predicted by AlphaFold2 or analogous tools - introduces even greater deviations from real values. We performed a brief test to compare docking results obtained from artificial and experimental protein structures, and found a significant difference between them (see the AlphaFold2 docking comparison chapter in the Supplementary). This also means that the model is expected to perform better on the proteins, existing in the BindingDB. Despite ESM’s ability to process any proteins, the DTA model is trained on a specific set of proteins. Moreover, since restrictions on the protein molecular weight and length were applied during the training dataset filtering, the model performance will be better on proteins ranging from 50 to 2500 amino acids.

Regarding the other limitations and capabilities of using the DTA model, the most computationally expensive component is the ESM-2 model. The computational resource requirements for the DrugForm-DTA model are determined by those of the ESM-2 model requirements. The DTA model is designed to work only with low-molecular-weight ligands, not peptides. This is caused, among other factors, by the limitation of the Chemformer model to the length of SMILES strings of up to 512 characters. The advantages of the developed DTA model include the ability to work with proteins containing mutations. In addition, although the DTA model was trained on canonical SMILES, the Chemformer model, which is used to generate SMILES embeddings, was pre-trained on SMILES with augmentation.

When evaluating the model performance, we observed that the choice of benchmarks is highly limited and may lead to interpretational challenges. For the Davis benchmark dataset (Figure S5), we found that nearly 3/4 of the records in the dataset (71 %) have a binding affinity of 10 *µ*M (pKi=5). The results of a preliminary analysis of the Davis dataset are presented in the “Davis benchmark issues” chapter in the Supplementary. We suggest that a high-quality benchmark dataset should be sufficiently large and diverse to provide a reliable estimate of the model accuracy. A procedure similar to the introduction of example weights in the BindingDB-based training dataset, described in this paper, can be used to select the most reliable examples. We propose that the reference dataset should include only protein-ligand complexes for which a large number of experimental measurements are available from multiple independent research groups and for which reported values are exact with low variability across measurements rather than a threshold. Our analysis indicates that the KIBA dataset (117,657 records) is significantly larger than the Davis dataset (25,772 records), and affinity values in KIBA are more evenly distributed compared to those in Davis.

A high-quality and large dataset is a major factor influencing model performance and its applicability to real-world cases. The BindingDB database is one of the largest sources of experimental affinity constant values, and we used it to prepare our training dataset. It is a raw database, which contains threshold values, missing fields, partial amino acid sequences, anomalously overrepresented values, and, approximately half of its protein-ligand pairs, multiple experimentally measured values. We grouped records for the same protein-ligand pair into groups and calculated the confidence interval of a single experimental measurement for each pair, obtaining an average value *CI*_*exp*_= 1.3 order of magnitude. We also calculated the spread between maximal and minimal values for each pair and obtained an average value of 0.9 orders (Figure S6). These values are substantial, prompting us to conduct further investigation into experimental affinity measurements. We found that existing measurement techniques often have low accuracy [68], and the reported values are strongly dependent on the experimental design and human factors [68], [69]. Also, in almost all cases authors do not specify the binding type. This implies that the actual precision of experimental measurements is lower than typically reported.

We analyzed the behavior of the DrugForm-DTA model across different ranges of affinity values and found that the model tends to overestimate low affinities and underestimate high ones, with low-affinity cases (pKi*<*5) being the most affected. However, we consider this issue minor because practically such affinity ranges are of limited practical relevance to researchers. We estimated the average confidence interval of the model predictions on a large dataset and found that our model achieves a confidence level comparable to that of a single experimental measurement (Fig. 5F). Thus, our model reaches a confidence level comparable to training dataset, supporting our proposition that the primary approach to computational prediction of drug-target affinity relies mainly on high quality data, while neural network architecture is less important.

Therefore, the main direction for improving the model performance is enhancing the quality of the training data by introducing new filtering and ranking criteria - such as the experimental laboratory, study date, and metadata — along with integrating data from additional sources. Additionally, careful ablation studies are needed using new encoders for ligands, as well as variations in the number of layers and their size. Another direction for improving the model performance involves benchmarking under different training–test splits. We conducted a similar study on the KIBA benchmark dataset comparing random, cold drug scaffold, cold target and our proposed split (see the “Training-test split issues” chapter in the Supplementary). However, larger-scale analyses of training-test splits on large experimental datasets such as BindingDB are still required. In addition, new and reliable validation methods are critically needed to develop high-quality DTA models.

## 5 Conclusion

We developed the DrugForm-DTA model to predict the binding affinity of protein-ligand complexes. Our results indicate very high accuracy on the KIBA and Davis benchmarks (top1/top2). The DrugForm-DTA model, trained on the refined BindingDB dataset, achieves a confidence level comparable to that of a single experimental measurement. The trained DTA model along with the benchmark models is freely available. We also publish the code required to run and retrain these models. The processed BindingDB dataset, including the training-test split and the building script, is also available, enabling others to use it for training their own models or constructing benchmarks. We compared DrugForm-DTA and molecular modeling approaches with experimental measurements and concluded that the accuracy of the trained neural network outperforms classical methods for assessing binding affinity between small ligands and proteins. Our molecular modeling results are also publicly available.

Finally, the DrugForm-DTA model is applicable to a wide range of problems and represents a highly promising approach for the discovery of novel therapeutic molecules across a broad spectrum of diseases.

## CRediT authorship contribution statement

**Nikolai Bugaev-Makarovskiy:** Writing – original draft, Methodology, Data curation. **Anna Tashchilova:** Writing – original draft, Visualization, Validation, Methodology, Investigation, Conceptualization. **Ivan Khokhlov:** Writing – original draft, Visualization, Validation, Software, Methodology, Investigation, Data curation, Conceptualization. **Olga Glushkova:** Writing – review & editing, Methodology. **Anton Keskinov:** Supervision. **Vladimir Yudin:** Supervision, Funding acquisition. **Veronika Skvortsova:** Resources. **Dmitry Svetlichnyy:** Writing – review & editing, Supervision, Project administration, Funding acquisition. **Sergey Yudin:** Supervision.

## Declaration of Competing Interest

The authors have declared no conflict of interest

## Data Availability

The data and the code accompanying this study are freely available. The code repository https://github.com/drugform/uniqsar contains the framework to train and benchmark models, and also to launch them for inference. Check the readme file to get detailed usage info and instructions to reproduce the steps of this work. The repository cannot serve big files, so they are placed at https://doi.org/10.5281/zenodo.14949569. Follow instructions at the code repository to merge the data into your cloned repository. Among other files, there are:• The files data/bindingdb/bindingdb.csv and data/bindingdb/bindingdb_test.csv are the cleaned BindingDB dataset we used to train and evaluate DrugForm-DTA. • The DrugForm-DTA model itself is stored at the models/bindingdb directory. • The selective_test_results.csv file is the selective test results for 127 protein-ligand pairs, containing target values from BindingDB, model predictions, and overall modeling score results. • The file data/bindingdb/pair_stats.tar.gz contains histograms of the distribution of affinity constant values for 296 individual protein-ligand pairs from BindingDB with 80th percentile values and confidence intervals plotted. • The files models/bindingdb/test • metrics/pKi.csv, models/bindingdb/test_metrics/pIC50.csv contain DTA model predictions compared to targets for the test dataset.
