# Supplementary material for: DrugForm-DTA: Towards real-world drug-target binding affinity model
Source: Comput Struct Biotechnol J. 2025 Sep 18;27:4106–20. doi: 10.1016/j.csbj.2025.09.023 (PMC12495441; doi:10.1016/j.csbj.2025.09.023)
Supplement: Supplementary file 1 — Supplementary material [file mmc1.pdf]

DrugForm-DTA: Supplementary

Training-test split issues

The default training-test split in machine learning is the naive (random) split. Unfortunately it does not suits well the DTA task, because the frequency distribution of proteins and ligand structures is far from uniform, and it is more challenging for the trained model to perform on rare proteins and ligand scaffolds. Using random split in DTA leads to optimistic test results and overestimating model quality, but not showing the real picture of model behavior on novel proteins and ligands. In order to solve that issue more specific training-test splits are commonly used in DTA.

The most basic split is cold target, where test part contains records with proteins, not included in train part with any other ligands. Cold target split is easy to implement, but does not reflect ligands manifold.

Cold drug split is a similar concept, but selects test records with ligands, not presented in the training set. It is also easy to implement, but is quite useless, because there are much more unique ligands than unique proteins, and a randomly selected test set is pretty much a cold drug split already. Also, simple cold drug split does not take into account the structural proximity of ligands [1]. That is why the drug scaffold split is much more challenging.

In drug scaffold split we do not select just unique ligand structures, but select structures with most rare molecular scaffolds. In order to implement this split, we first need to extract Murcko scaffold [2] from each ligand, then to build a sorted frequency table for each scaffold. The test set is populated with records, which ligands are most rare in the scaffold frequency table. This split is extremely challenging for the model, because the test ligands as much different to training ligands as possible. Examples of molecules with rare scaffolds are given at Figure S1.

While the Davis and KIBA benchmarks provide own training-test splits, in order to properly test the DrugForm-DTA model, we performed own split for the prepared BindingDB dataset. The dataset is huge, containing almost

Scaffold		Scaffold	
1	c1ccc(-c2ccc(B3OCCO3)cc2)cc1	6	O=C(NC1CCNCC1)c1cc(C2CC2)on1
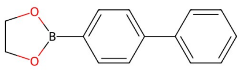	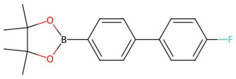	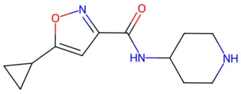	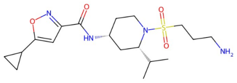
2	O=S(=O)(c1ccccc1)N1CCOc2ccccc21	7	O=C1C(=Cc2ccccc2)Oc2ccc3c(c21)OC1=CC=CC(=O)C13
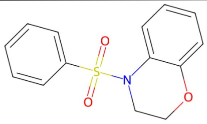	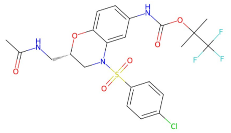	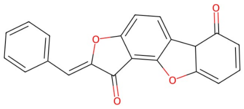	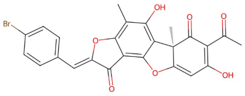
3	O=C(Nc1cccc(Nc2ncccc2-c2ncnc3[nH]ccc23)c1)c1ccccc1	8	O=C(CCCCCCc1ccccc1)c1ncco1
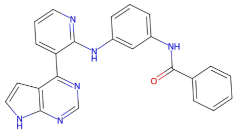	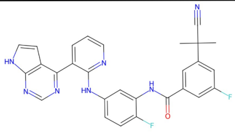	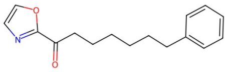	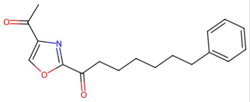
4	O=C1CCNC(=O)C(Cc2ccccc2)NC(=O)C(Cc2c[nH]c3ccccc23)NC(=O)CNC(=O)C(CC2CCCCC2)NC(=O)CN1	9	O=C(CCS(=O)(=O)Cc1ccc(OCc2ccccc2)cc1)N1CCCC1
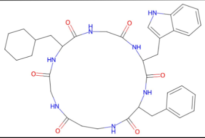	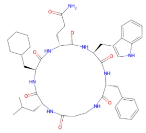	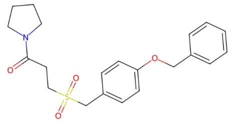	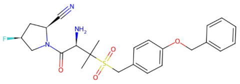
5	O=C1C=COS(=O)(=O)N1Cc1ccccc1	10	c1ccc2c(c1)OCCn1ccnc1-2
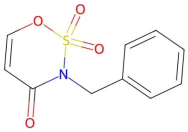	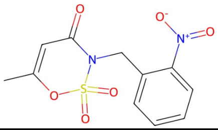	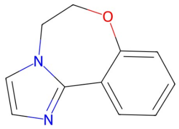	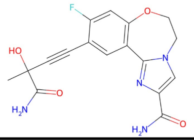

Figure S1: Examples of molecules with rare scaffolds.

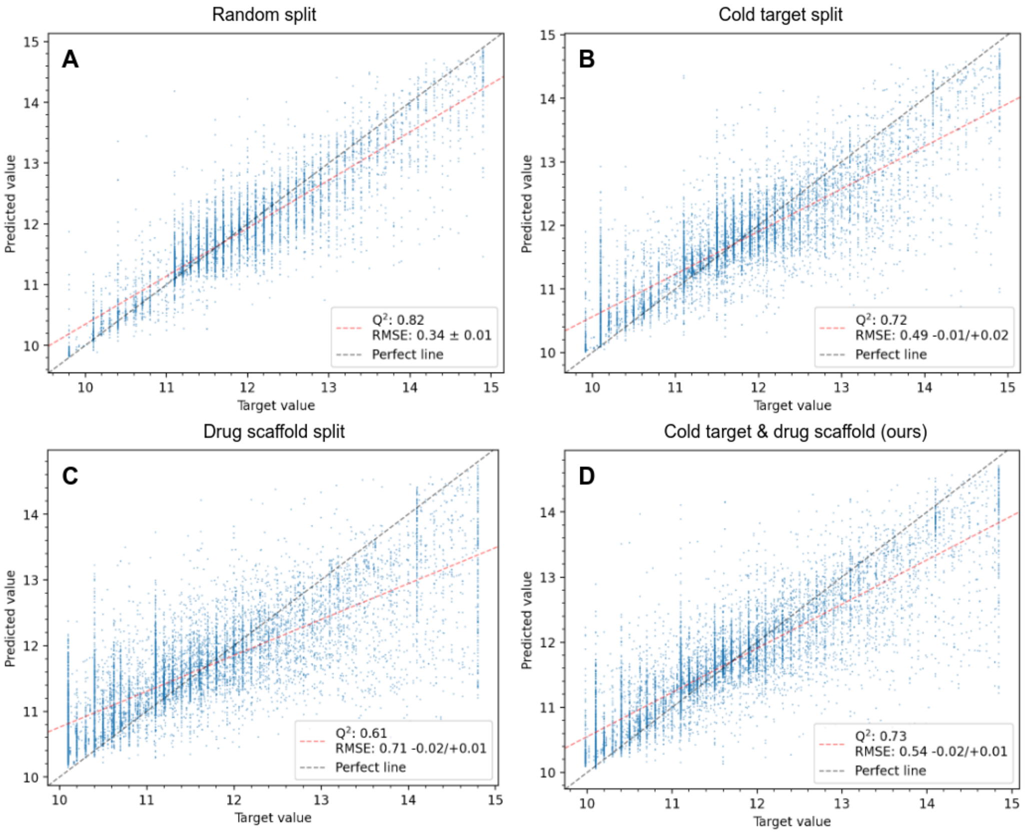

Figure S2: Test results on KIBA with different splits: A: Random split, B: Cold target split, C: Drug scaffold split, D: Our split.

2M records, and training multiple models with different splits is hard to afford. That's why we have chosen a single approach that combines drug- and target-based splits which we considered as a good default. The intended test set size is 5%, which is quite big in absolute numbers (91 000). First we use the cold target split approach to obtain one half of the test set, selecting 2.5% of all records. Then we used drug scaffold split to obtain the second half (another 2.5%) from the rest of BindingDB records. Thus our test set is a combination of two approaches, displaying how well the model performs on both.

In order to validate our split approach, we performed a low-scale experiment on KIBA dataset, comparing different split strategies: random, cold target, drug scaffold and ours. We applied different split strategies to the KIBA dataset and trained a separate model on each split. Training of each model took us 5 days. Scatter plots with test results for each model are at the Figure S2. Random split is expectably easiest with RMSE=0.34. Fully cold-target split gives RMSE=0.49, which is significantly larger than random split error. The most hard split at the KIBA dataset is drug scaffold split with RMSE=0.71. Obtaining 1/2 test set with target split and another 1/2 with drug scaffold split (our approach) gave RMSE=0.54, which is between cold target and cold drug scaffold splits.

This experiment proves that our split is something between two common strategies and can be used for DTA training.

SMILES robustness issues

One of the downsides of the SMILES notation is that the same compound can have several completely different SMILES representations. However, using the canonicalization procedure corrects this, but potentially leads to a generalization issue. In this regard, we conducted an additional study of using affinity model on augmented SMILES.

While inferring the SMILES string, RDKit traverses the molecular graph in the order given by CIP-ranks, assigned to each atom according to their importance. Taking away some atoms or inserting new ones can change CIP-ranks of existing atoms and thus change the graph traversal order. From this point SMILES is not robust: small changes in a molecule can lead to big changes in its representation. Still, the issue can be perfectly solved by training on SMILES-augmented data: for each data-point a random augmentation is used each time instead of the same canonical string.

Augmented string can be obtained by inferring SMILES string started from a random atom. ML models, trained that way, have proven to be robust (and usually give even better accuracy in QSAR).

To experimentally prove that we performed a test: we took a random single protein (UniProt ID: Q10469), which has more then 1000 ligand records in the training dataset. For each ligand from these 1000 we generated 10 random augmentations with RDKit and calculated standard deviation inside each group (separately for pKi and pIC50 values). An example of calculating standard deviation for a ligand and its augmented representations is in Table S1.

The distribution of standard deviation (std) values for each ligand is given in the Figure S3. Average std value is 0.26, meaning input augmented SMILES instead of canonical SMILES leads to average value deviation 0.26, which is 5 times lower than model confidence interval. Thus, even the model was not trained on augmented SMILES and is definitely overfitted at protein-ligand pairs from training set, it is robust to SMILES augmentation, which is a harder issue than permutations in canonical SMILES.

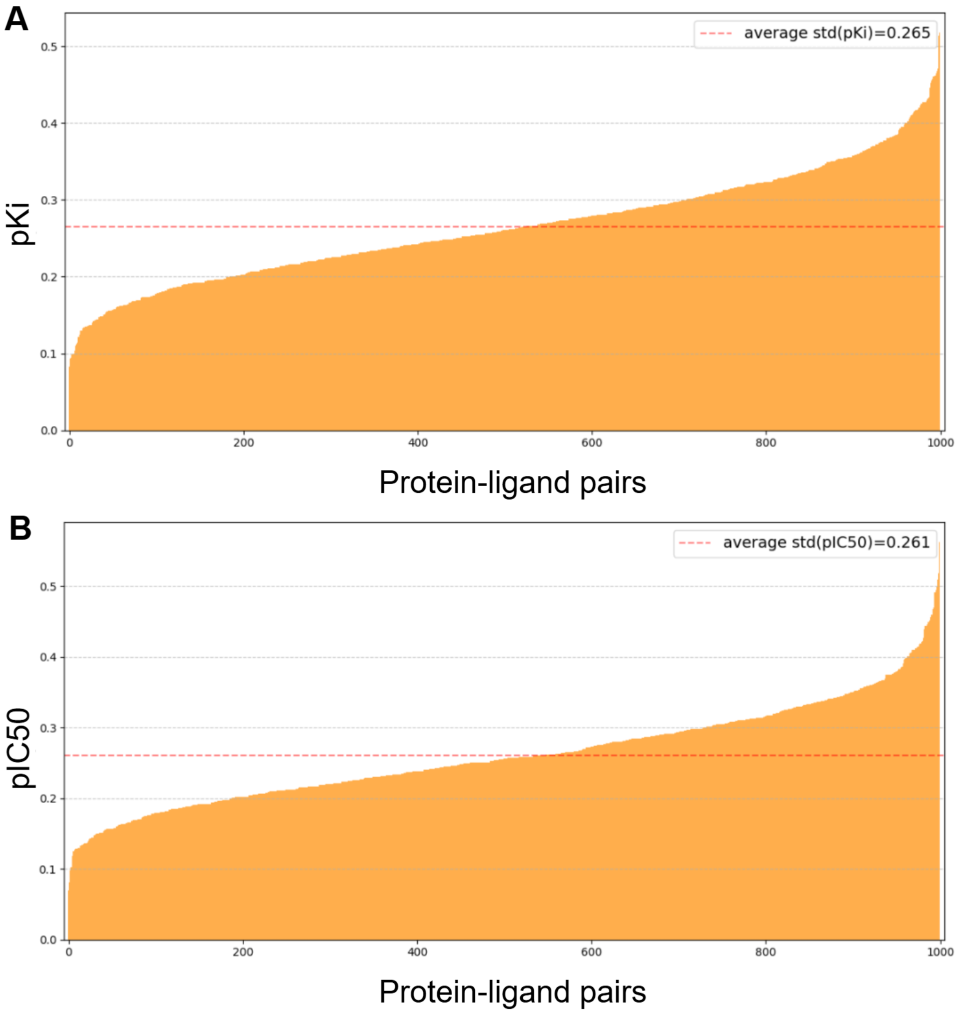

Figure S3: SMILES augmentation. A: pKi standard deviation distribution of augmented SMILES ligands B: pIC50 standard deviation distribution of augmented SMILES ligands.

Table S1: SMILES augmentation

**SMILES**	**pKi**	**pIC50**
CC(=O)NCC1=C(c2ccc(C(C)=O)cn2)C[C@](c2ccc (OCCCC(F)(F)F)cc2)(C(F)(F)F)NC1=O	7.1	7.0
C1(C[C@@](c2ccc(OCCCC(F)(F)F)cc2)(NC(=O) C=1CNC(C)=O)C(F)(F)F)c1ccc(C(C)=O)cn1	7.2	7.4
n1cc(C(=O)C)ccc1C1=C(C(N[C@@](C(F)(F)F)(C1) c1ccc(cc1)OCCCC(F)(F)F)=O)CNC(=O)C	7.1	7.1
C1(C(N[C@@](CC=1c1ccc(cn1)C(=O)C)(c1ccc (OCCCC(F)(F)F)cc1)C(F)(F)F)=O)CNC(=O)C	7.6	7.5
C(NC(C)=O)C1C(N[C@@](CC=1c1ccc(cn1)C(C)=O) (c1ccc(OCCCC(F)(F)F)cc1)C(F)(F)F)=O	7.4	7.3
c1c(C2C[C@@](C(F)(F)F)(c3ccc(OCCCC(F)(F)F)cc3) NC(C=2CNC(=O)C)=O)ncc(c1)C(=O)C	6.9	7.0
C1(=C(c2ccc(cn2)C(C)=O)C[C@](c2ccc(cc2)OCCCC (F)(F)F)(C(F)(F)F)NC1=O)CNC(C)=O	7.0	6.8
C1(=O)N[C@](C(F)(F)F)(c2ccc(OCCCC(F)(F)F)cc2) CC(c2ccc(C(C)=O)cn2)=C1CNC(C)=O	7.2	6.9
[C@]1(c2ccc(OCCCC(F)(F)F)cc2)(CC(=C(C(=O)N1) CNC(C)=O)c1ccc(cn1)C(=O)C)C(F)(F)F	7.1	7.2
c1cc(ccc1[C@]1(NC(=O)C(=C(C1)c1ncc(C(C)=O)cc1) CNC(C)=O)C(F)(F)F)OCCCC(F)(F)F	7.1	7.1
C1C(c2ccc(C(C)=O)cn2)=C(C(N[C@]1(C(F)(F)F) c1ccc(OCCCC(F)(F)F)cc1)=O)CNC(C)=O	7.1	7.1
mean	7.17	7.14
std	0.19	0.21

Calculating confidence intervals

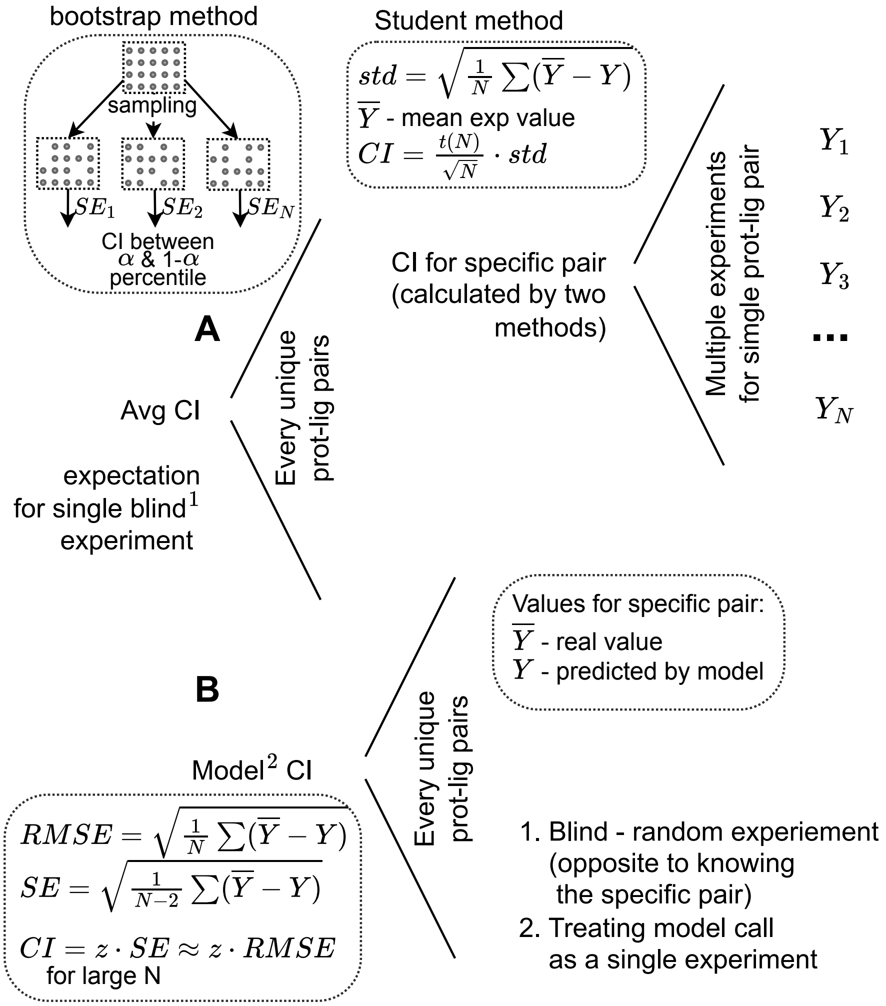

Figure S4: Calculating CI. A: Average CI for single experimental measurement. First calculating CI for every unique protein-ligand pair, then averaging over all pairs. Using both Student's and bootstrap approach. Student requires normal distribution assumption, but is able to work with very low-size sets. Bootstrapping does not require any assumptions about distribution, but tends to narrow the CI for low-size sets. Still they both gave the same result. B: Estimating model CI as z-score multiplied by standard error of estimate. For large N SE could be replaced with RMSE. Note that this is not the prediction interval (PI), which depends on specific input data.

Binding affinity experimental measurement issues

In modern biomedicine one of the key issues is the assessment of the potential interaction of substance A with substance B. Of particular interest is the assessment of the interaction between a low-molecular compound (ligand) and the target protein, since this is the cornerstone of pharmaceuticals and laboratory synthesis. In practice, we are most often interested not so much in the qualitative assessment of the interaction, but in its quantitative characteristics. For example, how many grams of ligand should be taken so that it fully interacts with the target protein for biochemical and functional changes in the organism or cell culture. The most correct method for quantitatively assessing the ligand-protein interaction is to assess the affinity between two compounds.

Affinity is usually understood as a quantitative (up to several decimal places) measurement of interaction strength between atoms and molecules of two substances that form a chemical bond or enter into a chemical interaction. Affinity in biochemistry is an assessment of the interaction between a ligand and a target molecule and reflects the amount of ligand substance (in nano- or picomoles) that will be necessary and sufficient to take for the interaction to occur and the desired effects to appear [3].

However, there is some confusion between the methods of integer display of affinity. The most commonly used method is the inhibition constant (Ki), which in most experiments is identical to the dissociation constant of the protein-ligand complex (Kd). But it is not uncommon to see the measurement of the half-maximal inhibitory concentration (IC50). For convenience of calculations, all values are usually converted to pKi/pIC50 (negative decimal logarithm of the value), rounded to 2 or 3 digits after the decimal point, which somewhat complicates direct interpretation of the data by a scientist during the experiment. As a result, a situation often arises where the same substance has Ki characteristics for binding to one protein and IC50 to another, or, conversely, a protein has several ligands, each with either only Ki or only IC50, while it is impossible to equate these two values without introducing coefficients obtained empirically for each protein-ligand pair separately [4, 5, 6].

The optimal solution is a huge study of the affinity of the ligand-protein pair empirically, which will allow obtaining a certain score of the Ki and/or IC50 values, with subsequent averaging or selection of several values depending on the reaction parameters. The empirical approach involves a

direct study of the interaction between the ligand and the protein in solution, followed by measuring the binding force (affinity) of the protein and ligand using computational practical methods. In particular, this can be a study using various methods of spectrometry (for example, UV spectroscopy or Fourier transform IR spectroscopy) [7], isothermal titration calorimetry [8], or microscopy (scanning electron microscopy or atomic force microscopy) [9]. Any of these methods has a measurement error (which consists of the error of the device and the errors made by the researcher), and also does not provide a complete picture of the binding of the ligand to the protein [10].

Moreover, a number of works allow us to evaluate a practical experiment as an experiment that always has a considerable margin of error [11, 10], while the magnitude of the error and its contribution to the final measurement depends primarily on the design of the experiment [11, 12]. For instance, we can look for experiments with simple vitamins like vitamin C (also known as ascorbic acid/ascorbate). Vitamin C is widely found in nature and foods, and is a popular food supplement - it is often used by people to prevent and treat common illnesses such as the common cold or flu. So, it's a common way to use tissue samples (f.i., peripheral blood) for any experiments with concentration of vitamin C like food additives or for investigation of interaction between vitamin C and any drug. Physiological plasma levels of vitamin C are 50–70 μM, but some organs and tissues have concentrations in the range from 1 mM (liver and lungs) to 10 mM (brain and adrenal glands). So, if we just take different cells (tissues) for equal *in vitro* experiments — we can discover differences about 10^2 [13]. And because of its levels of vitamin C in medical experiments (for example, clinical trials) very often chosen only by authors of paper. F.i., intravenously you can inject 50 mg/kg/24 (LoAscA) or 200 mg/kg/24 (HiAscA) [14]. Or you can use dose 1.5 g/patient without any corrections for weight/age [15]. Or also 1.5 g/patient, however, in different studies only the dosage of vitamin C is the same, but the timing of administration, the composition of the patient sample, the presence/absence of other drugs (for example, thiamine) and the effectiveness of therapy are completely different [16, 17].

And an increase in the number of point measurements may not only fail to solve the problem, but also increase the error if the experiment is designed incorrectly [18]. According to the literature, the affinity values (measured as Ki) are likely to be overestimated if there is an error during the experiment. This fact is explained by the nature of the measurements themselves and the human factor. Indeed, it is not uncommon for an experimenter to dilute or

titrate so that the point with the higher affinity is selected from the two values as the "last point at which Ki was measured", while in a number of methods rounding up can also play an important role [19, 18, 8]. At the same time, classical methods for assessing affinity often give rise to discussions about their applicability, because of a 10x dilution step is often used [5, 20]. For example, typical protocol for any antiviral research: “On the day of testing, the stock solution was subject to 10-fold serial dilutions with DMEM” [21]. Or typical way for antimicrobial research: “To assess the phloretin effect on S. mutans viability and determine MBC, 10-fold serial dilutions were made for each condition from the MIC microplate and 5 μl aliquots were spotted on BHI agar plate” [22]. It is also common practice, when researchers combine 10-fold and 2- or 3-fold dilution, because it is very comfortable to compare it. For instance, “a series dilution of substrates or inhibitors, such as EGCG (0, 1, 10, 100, 1000, 10000 μM; 10-fold dilution) or trypsin (0, 0.08, 0.25, 0.75, 2.25, 6.75 μM; 3-fold dilution)” [23]. Based on a common 10-fold dilution protocol we suggest converting threshold values into exact ones by shifting at 1/2 order in the direction of the threshold sign: “>1000nM” becomes 3160nM, which is between 1000nM and 10000nM.

From the point of view of the molecular mechanisms of the biochemical reaction, two factors are critical for the correct assessment of affinity. Firstly, this is the nature of the interaction of the ligand with the protein (at least, the binding is reversible or irreversible, in the case of inhibition, it is also critical whether it is competitive or non-competitive). And secondly, the correctness of the validation of the *in vitro* experiment in the *in vivo* experiment [6]. According to the literature, the nature of binding is not taken into account at all in most cases [6, 24, 25], and *in vivo* validation is rarely done and not always correctly, while revealing significant differences between cell culture and organism, as well as between organisms [26, 27]. All this, of course, introduces error into all datasets of experimental affinity data, which also complicates comparisons of one’s own experimentally obtained results with the data of colleagues.

A possible solution may be theoretical prediction of affinity (experiment *in silico*). In theoretical prediction the accuracy of the prediction depends both on the dataset on which we will build our hypotheses and on the modeling method. There are various databases of protein structures (for example, UniProt), and there are also specially prepared datasets of protein-ligand pairs with affinity values. However, as we found out earlier, the quality of the data itself most often raises questions and requires strict preliminary

filtering, otherwise all predictions will be incorrect regardless of the method. As for the modeling methods, here too it is worth highlighting critically important points.

Since the beginning of the century, spatial modeling methods such as docking and molecular dynamics have found wide application. However, 10 years ago it was shown that docking accuracy can vary from 0% to 92.66% depending on the selected protein, ligands and docking programs [28]. It is also widely known that the position of the ligand in spatial modeling depends primarily on the quality and completeness of the three-dimensional structure of the protein [28]. The growth of *in silico* affinity predictions has also led to a relatively large number of untested ligand-protein "bindings", many of which turn out to be not entirely accurate [29]. At the same time, a full-fledged study of the protein-ligand complex both *in silico* and *in vitro* requires huge amounts of substances and time costs, as well as a large amount of complex equipment [30], and is therefore carried out extremely rarely [10].

It is also important to mention the difficulties with the correct construction of mathematical hypotheses based on three-dimensional modeling. In fact, each experiment requires its own complex mathematical processing of data to identify the accuracy of the prediction in a given particular case [31]. Among the most correct, accessible and popular options for solving this problem is the use of the latest advances in machine learning and artificial intelligence, which allows us to significantly improve the accuracy of the forecast without increasing the time and resources spent on more complex and less correct studies using docking and molecular dynamics [32, 33, 34].

Thus, if we take into account all the controversial points, correctly filter the dataset for theoretical prediction, and conduct the *in silico* experiment using the most modern technologies of artificial intelligence and machine learning, it is possible to obtain a true prediction of affinity in a ligand-protein pair. With correct subsequent verification *in vitro* and validation *in vivo*, it will be possible not only to conduct a full-fledged study of the interaction of a ligand-protein pair, but also to improve our methods of theoretical prediction of affinity based on experimental data.

AlphaFold2 docking comparison

There are approaches to create artificial affinity datasets based on molecular docking [35, 36]. In this paper, we showed that docking correlates significantly lower with experimental values compared to a DTA model trained on real data. In addition, forming such a dataset requires spatial structures of target proteins, which are often obtained using AlphaFold.

We conducted a separate experiment dedicated to this problem, in which we compared the results of Glide molecular docking [37] using experimental X-ray protein structures and predicted AlphaFold protein structures. The advantage of using experimental protein structures compared to the AlphaFold2 [38] predicted ones was demonstrated using blood coagulation factor Xa and anticoagulants developed on the basis of its inhibitors. The experimental pKi affinity values were compared with DrugForm-DTA predictions and Glide docking scores obtained using the X-ray and AlphaFold2 target protein structures. We selected anticoagulants which have passed clinical trials and are approved for clinical usage as blood coagulation factor (FXa) ligands: apixaban (pKi=10), betrixaban (pKi=9.9), rivaroxaban (pKi=9.4) [39].

Comparison of the DrugForm-DTA predicted affinity values with the experimental ones showed a high correlation within the confidence interval. The Glide docking score using the experimental structure (PDB ID: 3CEN [40]) showed a much better correlation with the experimental values compared to the Glide docking score using the AlphaFold2 protein structure (Table S2). The docking score estimates the binding energy of the ligand to the protein, but it is not an affinity constant (pKi), so the absolute values of these quantities cannot be compared. Lower docking score means higher affinity. The docking score <-6.0 kcal/mol indicates the presence of an interaction between the ligand and the target protein. Betrixaban shows the most significant differences in the Glide docking scores (binding energy). The AlphaFold2 Glide docking score of -3.44 kcal/mol indicates no binding, which is inconsistent with the experimental measurements. At the same time, the PDB Glide docking score of -9.96 kcal/mol states an interaction of the ligand with the protein, consistent with the experimental data. The best agreement between the Glide docking score for both protein structures is observed for the apixaban, however, the usage of the predicted AlphaFold2 protein structure leads to an underestimation of the docking score (-7.13 kcal/mol AlphaFold2, -8.85 kcal/mol PDB structure). As a result of this

study, we state a reasonable doubt about suitability of using artificial data in DTA tasks.

Table S2: Glide docking results for three factor Xa inhibitor-based anticoagulants

Ligand Name	Experimental pKi	DrugForm-DTA Predicted pKi	AlphaFold2+Glide Energy, kcal/mol	PDB+Glide Energy, kcal/mol
Apixaban	10	9.1	-7.13	-8.85
Betrixaban	9.9	9.9	-3.44	-9.96
Rivaroxaban	9.4	8.8	-6.14	-7.8

Davis benchmark issues

The Davis dataset raises concerns about being a proper benchmark. First, we found out that 3/4 of its values are single value pKd=5. We decided to dig a little deeper.

Totally it contains 25772 records. We tried to find matching records in the original BindingDB database and found 13603 matches. We cannot say anything about other 12169 records except it is strange that they do not exist in one of the world's largest binding affinity database. From the 13603 matches 80% (10847) records have affinity value 10000 (pKd=5) in Davis, and most of them (99.1%) have the corresponding value ">10000" in BindingDB. This could mean that the Davis authors just replaced ">10000" with a strict 10000 value.

For the rest 94 matching record affinity values with "10000" value in Davis have simply different values in BindingDB, meaning 0.7% of Davis dataset values are just wrong. More specifically: 51 records have higher affinity (conflicting with ">10000" from Davis) and 43 records have lower affinity (not directly conflicts with ">10000" from Davis, but the authors could have used a known strict value).

Summing up, for 53% matches with BindingDB 80% records are unreliable, 0.7% are wrong, and non-matching records have to be a subject of a separate study. 3/4 records have same value, which is not good for regression benchmark.

Still, we have tested our model on it, because it is the basic DTA benchmark in community. At this point we do not claim that the Davis benchmark is broken, because such a statement requires a much deeper analysis, but we think that it should not be used without questioning.

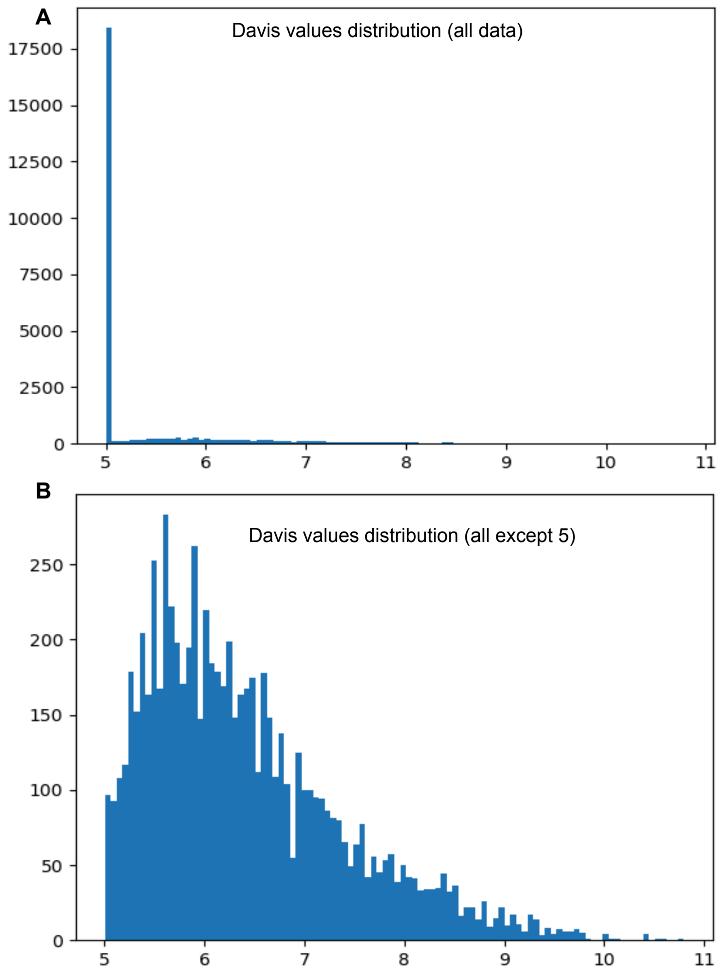

Figure S5: Davis dataset. A: pKd affinity values distribution in the Davis dataset. Almost 3/4 of all examples in the dataset have a single value of 5. Examples with other values are barely distinguishable in the histogram. B: pKd affinity values distribution in the Davis dataset when excluding data with pKd = 5. Examples with other values become visible, but the dataset size is reduced by three times.

Bindingdb value diffs for each protein-ligand complex

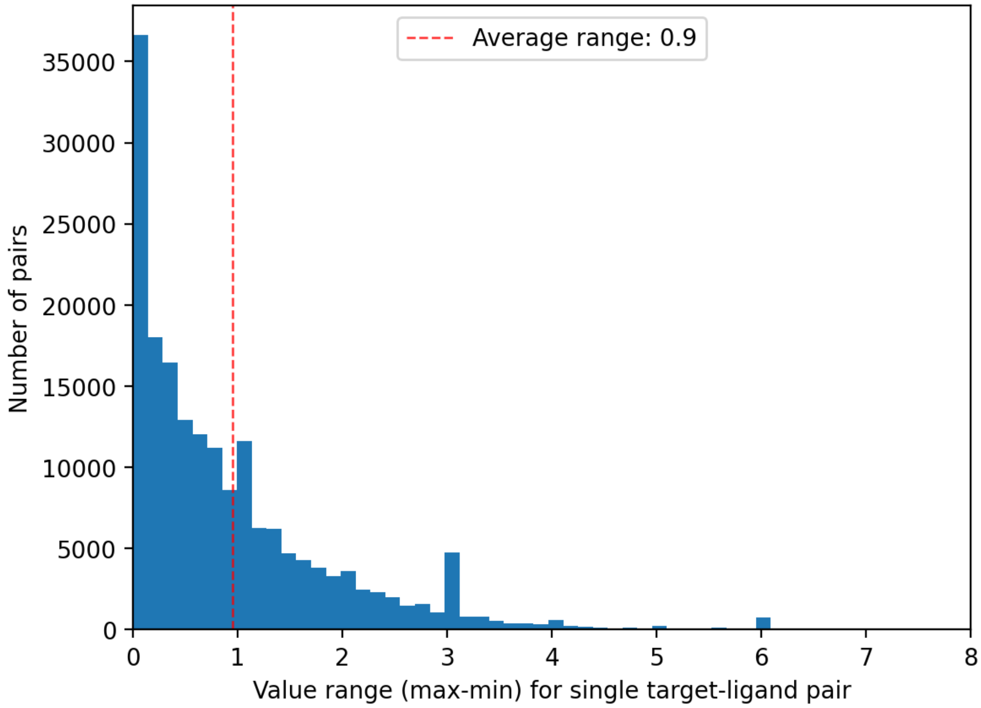

Figure S6: Value distribution for individual protein-ligand pairs in the original BindingDB dataset. The average spread is 0.9 orders of magnitude. There are examples with a spread of as much as 8 orders of magnitude. A significant number of pairs have zero spread, i.e. all measurements for a given pair have the same values. It is statistically unlikely that two independent experiments will give exactly the same value. These situations are presumably due to either duplication of the same result or rough rounding. If these records are excluded from the spread estimate, the average spread will increase.

Molecular modeling

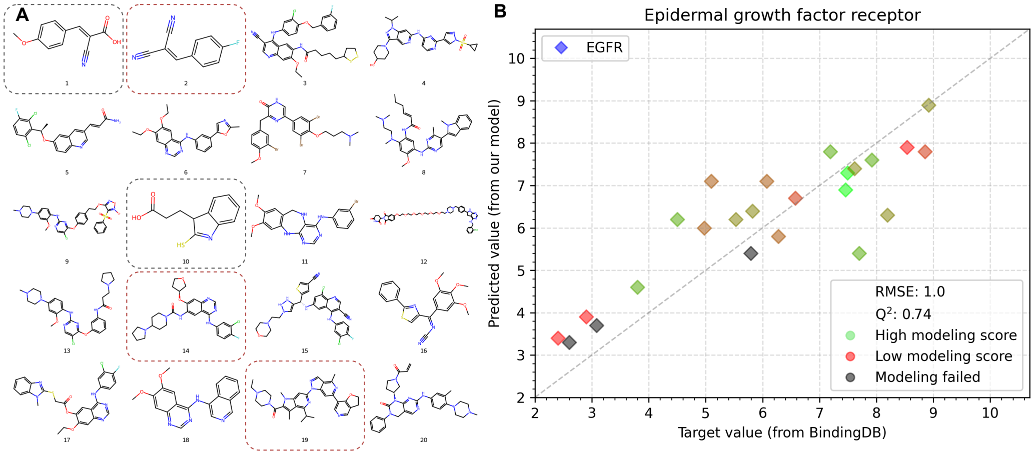

Figure S7: Molecular modeling EGFR. A: 2D structures of 20 ligands for EGFR used for the selective test. Molecules with low modeling scores are marked red, and grey means modeling failed. B: Scatter plot visualizes the correlations among experimental values, DrugForm-DTA predicted values and modeling scores. All modeling failed protein-ligand complexes marked grey and related to EGFR protein (C_{Mod}(EGFR)=0.18, C_{DTA}(EGFR)=0.80).

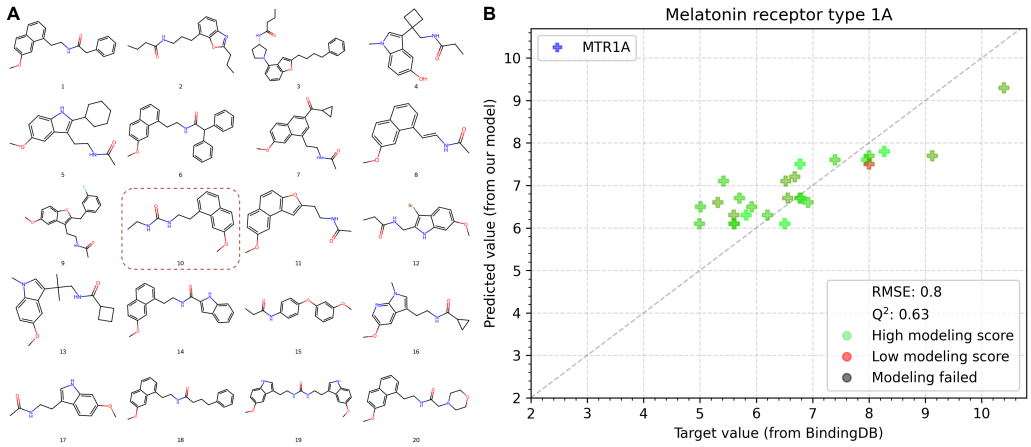

Figure S8: Molecular modeling MTR1A. A: 2D structures of 20 ligands for MTR1A used for the selective test. Molecules with low modeling scores are marked red. B: Scatter plot visualizes the correlations among experimental values, DrugForm-DTA predicted values and modeling scores. Modeling of MTR1A binding revealed high modeling score values for almost all ligands from the test set, even for those with lower experimental values, that leads to low correlation values (C_{Mod}(MTR1A)=-0.03, C_{DTA}(MTR1A)=0.80). Thus, molecular scores are poorly suitable to distinguish between high and medium affinities (pKi/pIC50 in range from 5 to 8).

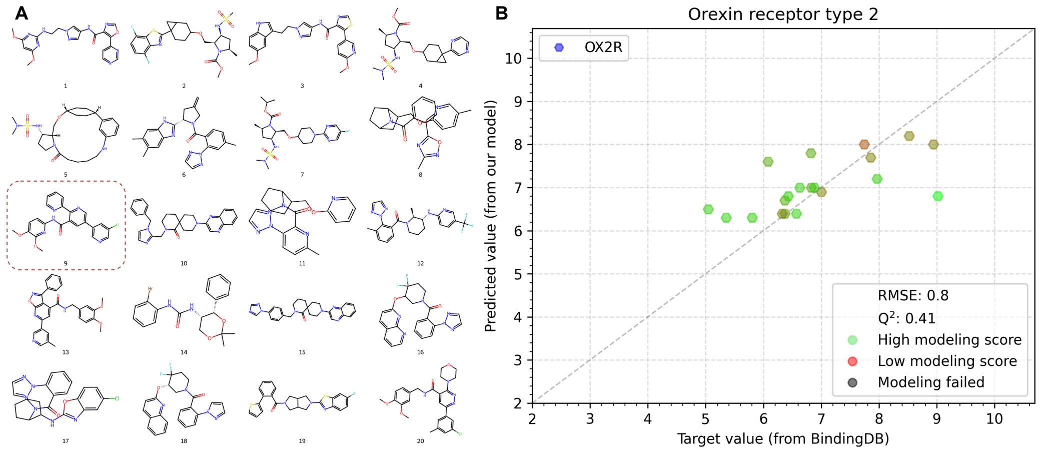

Figure S9: Molecular modeling OX2R. A: 2D structures of 20 ligands for OX2R used for the selective test. Molecules with low modeling scores are marked red. B: Scatter plot visualizes the correlations among experimental values, DrugForm-DTA predicted values and modeling scores. Modeling of OX2R binding revealed high modeling score values for almost all ligands from the test set, even for those with lower experimental values, that leads to low correlation values (C_{Mod}(OX2R)=-0.26, C_{DTA}(OX2R)=0.70). Thus, molecular scores are poorly suitable to distinguish between high and medium affinities (pKi/pIC50 in range from 5 to 8).

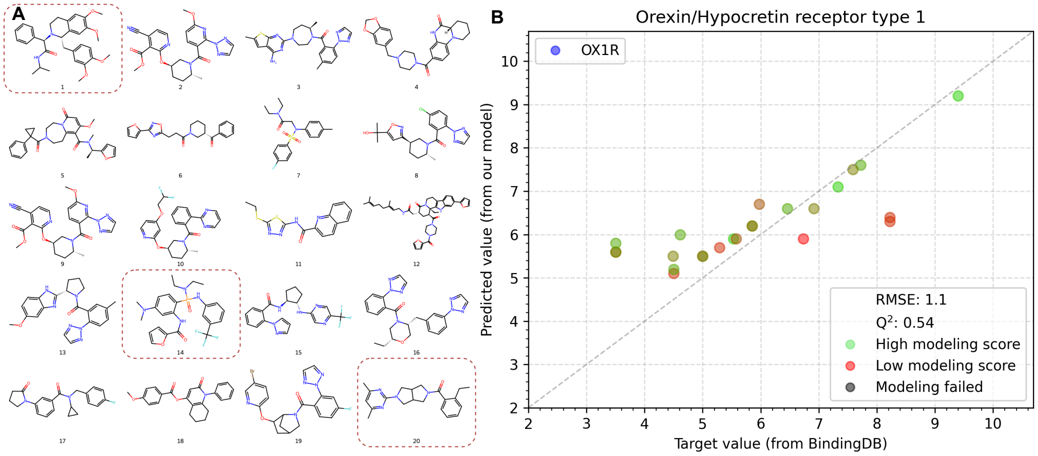

Figure S10: Molecular modeling OX1R. A: 2D structures of 20 ligands for OX1R used for the selective test. Molecules with low modeling scores are marked red. B: Scatter plot visualizes the correlations among experimental values, DrugForm-DTA predicted values and modeling scores. Modeling of ligands binding with OX1R (C_{Mod}(OX1R)=-0.04, C_{DTA}(OX1R)=0.84) shows no correlation with experiment.

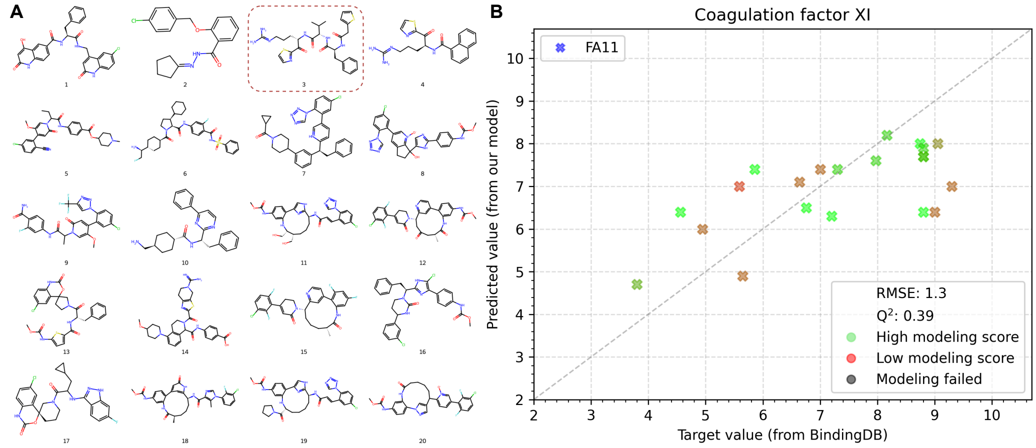

Figure S11: Molecular modeling FA11. A: 2D structures of 20 ligands for FA11 used for the selective test. Molecules with low modeling scores are marked red. B: Scatter plot visualizes the correlations among experimental values, DrugForm-DTA predicted values and modeling scores. For FA11 (C_{Mod}(FA11)=0.06, C_{DTA}(FA11)=0.55) the modeling scores do not fully correlate with experiment, that is supposedly caused by missing a significant part of the amino acid residues of the light chain.

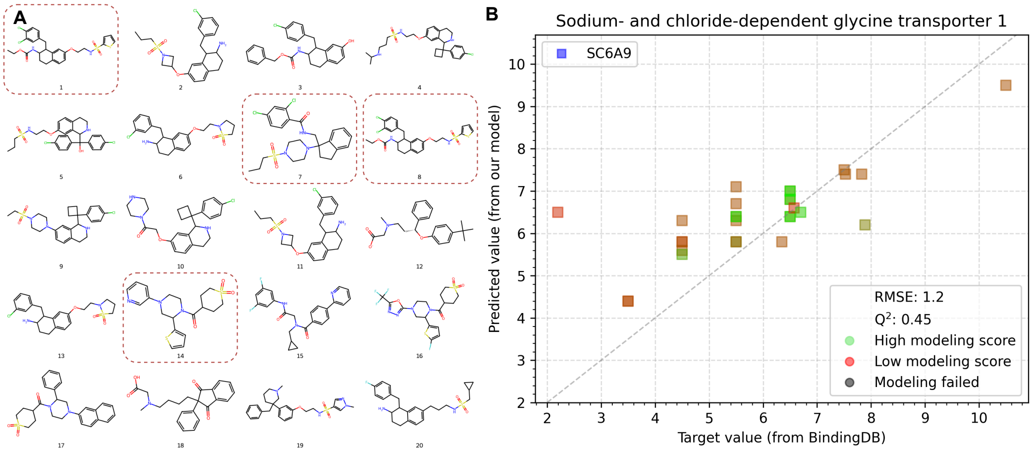

Figure S12: Molecular modeling SC6A9. A: 2D structures of 20 ligands for SC6A9 used for the selective test. Molecules with low modeling scores are marked red. B: Scatter plot visualizes the correlations among experimental values, DrugForm-DTA predicted values and modeling scores. A large number of ligands with low modeling scores are observed for protein–ligand complexes with SC6A9 (C_{Mod}(SC6A9)=0.27, C_{DTA}(SC6A9)=0.70).

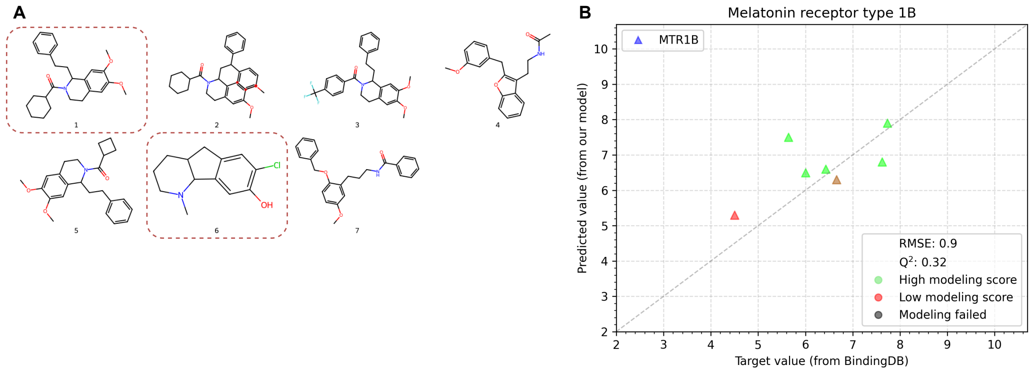

Figure S13: Molecular modeling MTR1B. A: 2D structures of 7 ligands for MTR1B used for the selective test. Molecules with low modeling scores are marked red. B: Scatter plot visualizes the correlations among experimental values, DrugForm-DTA predicted values and modeling scores. A large number of ligands with low modeling scores are observed for protein–ligand complexes with MTR1B (C_{Mod}(MTR1B)=0.40, C_{DTA}(MTR1B)=0.54).

References

[1] T. M. Nguyen, T. Nguyen, T. Tran, Mitigating cold-start problems in drug-target affinity prediction with interaction knowledge transferring, Briefings in Bioinformatics 23 (4) (2022) bbac269, _eprint: https://academic.oup.com/bib/article-pdf/23/4/bbac269/45267785/bbac269.pdf. doi:10.1093/bib/bbac269. URL https://doi.org/10.1093/bib/bbac269

[2] G. W. Bemis, M. A. Murcko, The properties of known drugs. 1. molecular frameworks, Journal of medicinal chemistry 39 (15) (1996) 2887–2893.

[3] M. Eddy, Elements, Principles and the Narrative of Affinity, Foundations of Chemistry 6 (2) (2004) 161–175. doi:10.1023/B:FOCH.0000035061.02831.45. URL http://link.springer.com/10.1023/B:FOCH.0000035061.02831.45

[4] B. Srinivasan, M. D. Lloyd, Dose–Response Curves and the Determination of IC_{50} and EC_{50} Values, Journal of Medicinal Chemistry 67 (20) (2024) 17931–17934. doi:10.1021/acs.jmedchem.4c02052. URL https://pubs.acs.org/doi/10.1021/acs.jmedchem.4c02052

[5] S. R. Hoare, The Problems of Applying Classical Pharmacology Analysis to Modern In Vitro Drug Discovery Assays: Slow Binding Kinetics and High Target Concentration, SLAS Discovery 26 (7) (2021) 835–850. doi:10.1177/24725552211019653. URL https://linkinghub.elsevier.com/retrieve/pii/S2472555222067351

[6] B.-F. Krippendorff, R. Neuhaus, P. Lienau, A. Reichel, W. Huisinga, Mechanism-Based Inhibition: Deriving KI and kinact Directly from Time-Dependent IC50 Values, SLAS Discovery 14 (8) (2009) 913–923. doi:10.1177/1087057109336751. URL https://linkinghub.elsevier.com/retrieve/pii/S2472555222081175

[7] Y. Meng, Z. Wei, C. Xue, Deciphering the interaction mechanism and binding mode between chickpea protein isolate and flavonoids based on experimental studies and molecular simulation, Food Chemistry 429 (2023) 136848. doi:10.1016/j.foodchem.2023.136848. URL https://linkinghub.elsevier.com/retrieve/pii/S0308814623014668

[8] M. Bastos, O. Abian, C. M. Johnson, F. Ferreira-da Silva, S. Vega, A. Jimenez-Alesanco, D. Ortega-Alarcon, A. Velazquez-Campoy, Isothermal titration calorimetry, Nature Reviews Methods Primers 3 (1) (2023) 17. doi:10.1038/s43586-023-00199-x.
URL https://www.nature.com/articles/s43586-023-00199-x

[9] R. Wang, Y. Wang, Z. Lei, L. Hao, L. Jiang, Glucosyltransferase-modulated *Streptococcus mutans* adhesion to different surfaces involved in biofilm formation by atomic force microscopy, Microbiology and Immunology 66 (11) (2022) 493–500. doi:10.1111/1348-0421.13025.
URL
https://onlinelibrary.wiley.com/doi/10.1111/1348-0421.13025

[10] Z. Jin, Z. Wei, Molecular simulation for food protein–ligand interactions: A comprehensive review on principles, current applications, and emerging trends, Comprehensive Reviews in Food Science and Food Safety 23 (1) (2024) e13280. doi:10.1111/1541-4337.13280.
URL https://ift.onlinelibrary.wiley.com/doi/10.1111/1541-4337.13280

[11] M. Stein, R. Haselberg, M. Mozafari-Torshizi, H. Wätzig, Experimental design and measurement uncertainty in ligand binding studies by affinity capillary electrophoresis, ELECTROPHORESIS 40 (7) (2019) 1041–1054. doi:10.1002/elps.201800450.
URL https://analyticalsciencejournals.onlinelibrary.wiley.com/doi/10.1002/elps[illegible]

[12] N. Georgakis, E. Ioannou, C. Varotsou, G. Premetis, E. G. Chronopoulou, N. E. Labrou, Determination of Half-Maximal Inhibitory Concentration of an Enzyme Inhibitor, in: N. E. Labrou (Ed.), Targeting Enzymes for Pharmaceutical Development, Vol. 2089, Springer US, New York, NY, 2020, pp. 41–46, series Title: Methods in Molecular Biology. doi:10.1007/978-1-0716-0163-1_3.
URL http://link.springer.com/10.1007/978-1-0716-0163-1_3

[13] J. Lykkesfeldt, P. Tveden-Nyborg, The pharmacokinetics of vitamin c, Nutrients 11 (10) (2019). doi:10.3390/nu11102412.
URL https://www.mdpi.com/2072-6643/11/10/2412

[14] A. A. Fowler, A. A. Syed, S. Knowlson, R. Sculthorpe, D. Farthing, C. DeWilde, C. A. Farthing, T. L. Larus, E. Martin, D. F. Brophy, S. Gupta, Medical Respiratory Intensive Care Unit Nursing, B. J. Fisher,

R. Natarajan, Phase i safety trial of intravenous ascorbic acid in patients with severe sepsis, Journal of Translational Medicine 12 (1) (2014) 32. doi:10.1186/1479-5876-12-32.
URL `https://doi.org/10.1186/1479-5876-12-32`

[15] C. J. Lindsell, A. McGlothlin, S. Nwosu, T. W. Rice, A. Hall, G. R. Bernard, L. W. Busse, E. W. Ely, A. A. Fowler, D. F. Gaieski, J. S. Hinson, M. H. Hooper, J. C. Jackson, G. D. Kelen, M. Levine, G. S. Martin, R. E. Rothman, J. E. Sevransky, K. Viele, D. W. Wright, D. N. Hager, Update to the vitamin c, thiamine and steroids in sepsis (victas) protocol: statistical analysis plan for a prospective, multicenter, double-blind, adaptive sample size, randomized, placebo-controlled, clinical trial, Trials 20 (1) (2019) 670. doi:10.1186/s13063-019-3775-8.
URL `https://doi.org/10.1186/s13063-019-3775-8`

[16] C. Vollbracht, K. Kraft, Oxidative stress and hyper-inflammation as major drivers of severe covid-19 and long covid: Implications for the benefit of high-dose intravenous vitamin c, Frontiers in Pharmacology Volume 13 - 2022 (2022). doi:10.3389/fphar.2022.899198.
URL `https://www.frontiersin.org/journals/pharmacology/articles/10.3389/fphar.`[illegible]

[17] S. S. Scholz, R. Borgstedt, N. Ebeling, L. C. Menzel, G. Jansen, S. Rehberg, Mortality in septic patients treated with vitamin c: a systematic meta-analysis, Critical Care 25 (1) (2021) 17. doi:10.1186/s13054-020-03438-9.
URL `https://doi.org/10.1186/s13054-020-03438-9`

[18] D. J. Murphy, Determination of accurate KI values for tight-binding enzyme inhibitors: an in silico study of experimental error and assay design, Analytical Biochemistry 327 (1) (2004) 61–67. doi:10.1016/j.ab.2003.12.018.
URL `https://linkinghub.elsevier.com/retrieve/pii/S0003269704000351`

[19] M. L. Reytor Gonzalez, M. Alonso Del Rivero Antigua, Reviewing the experimental and mathematical factors involved in tight binding inhibitors Ki values determination: The bi-functional protease inhibitor SmCI as a test model, Biochimie 181 (2021) 86–95. doi:10.1016/j.biochi.2020.11.014.
URL `https://linkinghub.elsevier.com/retrieve/pii/S0300908420303023`

[20] T. Ren, X. Zhu, N. M. Jusko, W. Krzyzanski, W. J. Jusko, Pharmacodynamic model of slow reversible binding and its applications in pharmacokinetic/pharmacodynamic modeling: review and tutorial, Journal of Pharmacokinetics and Pharmacodynamics 49 (5) (2022) 493–510. doi:10.1007/s10928-022-09822-y.
URL `https://link.springer.com/10.1007/s10928-022-09822-y`

[21] W. Shang, W. Dai, C. Yao, L. Xu, X. Tao, H. Su, J. Li, X. Xie, Y. Xu, M. Hu, D. Xie, H. Jiang, L. Zhang, H. Liu, In vitro and in vivo evaluation of the main protease inhibitor fb2001 against sars-cov-2, Antiviral Research 208 (2022) 105450. doi:https://doi.org/10.1016/j.antiviral.2022.105450.
URL `https://www.sciencedirect.com/science/article/pii/S0166354222002194`

[22] L. Rudin, M. M. Bornstein, V. Shyp, Inhibition of biofilm formation and virulence factors of cariogenic oral pathogen streptococcus mutans by natural flavonoid phloretin, Journal of Oral Microbiology 15 (1) (2023) 2230711, pMID: 37416858. arXiv:https://doi.org/10.1080/20002297.2023.2230711, doi:10.1080/20002297.2023.2230711.
URL `https://doi.org/10.1080/20002297.2023.2230711`

[23] Z. Zeng, C. Lin, C. Pan, Z. Chen, B. H. Ruan, An alternative mechanism of glutamate dehydrogenase inhibition by egcg: Promotion of protein degradation, Pharmaceuticals 18 (6) (2025). doi:10.3390/ph18060877.
URL `https://www.mdpi.com/1424-8247/18/6/877`

[24] L. Boike, N. J. Henning, D. K. Nomura, Advances in covalent drug discovery, Nature Reviews Drug Discovery 21 (12) (2022) 881–898. doi:10.1038/s41573-022-00542-z.
URL `https://www.nature.com/articles/s41573-022-00542-z`

[25] E. Mons, S. Roet, R. Q. Kim, M. P. C. Mulder, A Comprehensive Guide for Assessing Covalent Inhibition in Enzymatic Assays Illustrated with Kinetic Simulations, Current Protocols 2 (6) (2022) e419. doi:10.1002/cpz1.419.
URL `https://currentprotocols.onlinelibrary.wiley.com/doi/10.1002/cpz1.419`

[26] A. Mohammadinejad, T. Mohajeri, G. Aleyaghoob, F. Heidarian, R. Kazemi Oskuee, Ellagic acid as a potent anticancer drug: A

comprehensive review on in vitro, in vivo, in silico, and drug delivery studies, Biotechnology and Applied Biochemistry 69 (6) (2022) 2323–2356. doi:10.1002/bab.2288.
URL
https://iubmb.onlinelibrary.wiley.com/doi/10.1002/bab.2288

[27] A. Awadasseid, R. Wang, S. Sun, F. Zhang, Y. Wu, W. Zhang, Small molecule and PROTAC molecule experiments in vitro and in vivo, focusing on mouse PD-L1 and human PD-L1 differences as targets, Biomedicine & Pharmacotherapy 172 (2024) 116257. doi:10.1016/j.biopha.2024.116257.
URL https://linkinghub.elsevier.com/retrieve/pii/S0753332224001380

[28] Y.-C. Chen, Beware of docking!, Trends in Pharmacological Sciences 36 (2) (2015) 78–95, publisher: Elsevier. doi:10.1016/j.tips.2014.12.001.
URL https://doi.org/10.1016/j.tips.2014.12.001

[29] H. Nada, N. A. Meanwell, M. T. Gabr, Virtual screening: hope, hype, and the fine line in between, Expert Opinion on Drug Discovery 20 (2) (2025) 145–162. doi:10.1080/17460441.2025.2458666.
URL https://www.tandfonline.com/doi/full/10.1080/17460441.2025.2458666

[30] P. Serafin, L. Szeleszczuk, I. Zhukov, E. Szűcs, D. Gombos, A. Stefanucci, A. Mollica, D. M. Pisklak, P. Kleczkowska, Opioid/Dopamine Receptor Binding Studies, NMR and Molecular Dynamics Simulation of LENART01 Chimera, an Opioid-Bombesin-like Peptide, Molecules 29 (1) (2024) 272. doi:10.3390/molecules29010272.
URL https://www.mdpi.com/1420-3049/29/1/272

[31] R. F. Staack, G. Jordan, J. Heinrich, Mathematical Simulations For Bioanalytical Assay Development: The (Un-)Necessity And (Im-)Possibility of Free Drug Quantification, Bioanalysis 4 (4) (2012) 381–395. doi:10.4155/bio.11.321.
URL
https://www.tandfonline.com/doi/full/10.4155/bio.11.321

[32] L. Zhang, C.-C. Wang, Y. Zhang, X. Chen, GPCNDTA: Prediction of drug-target binding affinity through cross-attention networks augmented with graph features and pharmacophores, Computers in Biology and

Medicine 166 (2023) 107512. doi:10.1016/j.compbiomed.2023.107512.
URL https://linkinghub.elsevier.com/retrieve/pii/S0010482523009770

[33] N. Arul Murugan, G. Ruba Priya, G. Narahari Sastry, S. Markidis, Artificial intelligence in virtual screening: Models versus experiments, Drug Discovery Today 27 (7) (2022) 1913–1923. doi:10.1016/j.drudis.2022.05.013.
URL https://linkinghub.elsevier.com/retrieve/pii/S1359644622002033

[34] P. C. Tiwari, R. Pal, M. J. Chaudhary, R. Nath, Artificial intelligence revolutionizing drug development: Exploring opportunities and challenges, Drug Development Research 84 (8) (2023) 1652–1663. doi:10.1002/ddr.22115.
URL https://onlinelibrary.wiley.com/doi/10.1002/ddr.22115

[35] Y. Z. Zhang, CrossDocked2020. doi:10.21227/45C9-VG74.
URL https://ieee-dataport.org/documents/crossdocked2020

[36] A. A. Sadybekov, A. V. Sadybekov, Y. Liu, C. Iliopoulos-Tsoutsouvas, X.-P. Huang, J. Pickett, B. Houser, N. Patel, N. K. Tran, F. Tong, N. Zvonok, M. K. Jain, O. Savych, D. S. Radchenko, S. P. Nikas, N. A. Petasis, Y. S. Moroz, B. L. Roth, A. Makriyannis, V. Katritch, Synthon-based ligand discovery in virtual libraries of over 11 billion compounds, Nature 601 (7893) (2022) 452–459. doi:10.1038/s41586-021-04220-9.
URL https://www.nature.com/articles/s41586-021-04220-9

[37] R. A. Friesner, J. L. Banks, R. B. Murphy, T. A. Halgren, J. J. Klicic, D. T. Mainz, M. P. Repasky, E. H. Knoll, M. Shelley, J. K. Perry, D. E. Shaw, P. Francis, P. S. Shenkin, Glide: A New Approach for Rapid, Accurate Docking and Scoring. 1. Method and Assessment of Docking Accuracy, Journal of Medicinal Chemistry 47 (7) (2004) 1739–1749, _eprint: https://doi.org/10.1021/jm0306430. doi:10.1021/jm0306430.
URL https://doi.org/10.1021/jm0306430

[38] J. Jumper, R. Evans, A. Pritzel, T. Green, M. Figurnov, O. Ronneberger, K. Tunyasuvunakool, R. Bates, A. Žídek, A. Potapenko, A. Bridgland, C. Meyer, S. A. A. Kohl, A. J. Ballard, A. Cowie, B. Romera-Paredes, S. Nikolov, R. Jain, J. Adler, T. Back, S. Petersen, D. Reiman, E. Clancy, M. Zielinski, M. Steinegger, M. Pacholska, T. Berghammer, S. Bodenstein, D. Silver, O. Vinyals,

A. W. Senior, K. Kavukcuoglu, P. Kohli, D. Hassabis, Highly accurate protein structure prediction with AlphaFold, Nature 596 (7873) (2021) 583–589. doi:10.1038/s41586-021-03819-2.

[39] F. Siddiqui, D. Hoppensteadt, W. Jeske, O. Iqbal, A. Tafur, J. Fareed, Factor Xa Inhibitory Profile of Apixaban, Betrixaban, Edoxaban, and Rivaroxaban Does Not Fully Reflect Their Biologic Spectrum, Clinical and Applied Thrombosis/Hemostasis 25 (2019) 1076029619847524. doi:10.1177/1076029619847524.
URL
`https://journals.sagepub.com/doi/10.1177/1076029619847524`

[40] H. M. Berman, J. Westbrook, Z. Feng, G. Gilliland, T. N. Bhat, H. Weissig, I. N. Shindyalov, P. E. Bourne, The Protein Data Bank, Nucleic Acids Research 48 (2000) 235–242. doi:https://doi.org/10.1093/nar/28.1.235. URL `http://www.rcsb.org/pdb/`
